# Evolution of enhanced innate immune evasion by SARS-CoV-2

**DOI:** 10.1038/s41586-021-04352-y

**Published:** 2021-12-23

**Authors:** Lucy G. Thorne, Mehdi Bouhaddou, Ann-Kathrin Reuschl, Lorena Zuliani-Alvarez, Ben Polacco, Adrian Pelin, Jyoti Batra, Matthew V. X. Whelan, Myra Hosmillo, Andrea Fossati, Roberta Ragazzini, Irwin Jungreis, Manisha Ummadi, Ajda Rojc, Jane Turner, Marie L. Bischof, Kirsten Obernier, Hannes Braberg, Margaret Soucheray, Alicia Richards, Kuei-Ho Chen, Bhavya Harjai, Danish Memon, Joseph Hiatt, Romel Rosales, Briana L. McGovern, Aminu Jahun, Jacqueline M. Fabius, Kris White, Ian G. Goodfellow, Yasu Takeuchi, Paola Bonfanti, Kevan Shokat, Natalia Jura, Klim Verba, Mahdad Noursadeghi, Pedro Beltrao, Manolis Kellis, Danielle L. Swaney, Adolfo García-Sastre, Clare Jolly, Greg J. Towers, Nevan J. Krogan

**Affiliations:** 1 Present Address: Division of Infection and Immunity, University College London, London, UK; 2 QBI Coronavirus Research Group (QCRG), University of California San Francisco, San Francisco, CA USA; 3 Quantitative Biosciences Institute (QBI), University of California San Francisco, San Francisco, CA USA; 4 J. David Gladstone Institutes, San Francisco, CA USA; 5 Department of Cellular and Molecular Pharmacology, University of California San Francisco, San Francisco, CA USA; 6 Division of Virology, Department of Pathology, Addenbrooke’s Hospital, University of Cambridge, Cambridge, UK; 7 Epithelial Stem Cell Biology and Regenerative Medicine Laboratory, The Francis Crick Institute, London, UK; 8 MIT Computer Science and Artificial Intelligence Laboratory, MIT, Cambridge, MA USA; 9 Broad Institute of MIT and Harvard, Cambridge, MA USA; 10 European Molecular Biology Laboratory (EMBL), European Bioinformatics Institute, Wellcome Genome Campus, Hinxton, UK; 11 Department of Microbiology, Icahn School of Medicine at Mount Sinai, New York, NY USA; 12 Global Health and Emerging Pathogens Institute, Icahn School of Medicine at Mount Sinai, New York, NY USA; 13 Howard Hughes Medical Institute, San Francisco, CA USA; 14 Division of Advanced Therapies, National Institute for Biological Standards and Control, South Mimms, UK; 15 Present Address: Cardiovascular Research Institute, University of California San Francisco, San Francisco, CA USA; 16 The Tisch Cancer Institute, Icahn School of Medicine at Mount Sinai, New York, NY USA; 17 Department of Medicine, Division of Infectious Diseases, Icahn School of Medicine at Mount Sinai, New York, NY USA; 18 Department of Pathology, Molecular and Cell-Based Medicine, Icahn School of Medicine at Mount Sinai, New York, NY USA

**Keywords:** SARS-CoV-2, Systems biology

## Abstract

The emergence of SARS-CoV-2 variants of concern suggests viral adaptation to enhance human-to-human transmission^1,2^. Although much effort has focused on the characterization of changes in the spike protein in variants of concern, mutations outside of spike are likely to contribute to adaptation. Here, using unbiased abundance proteomics, phosphoproteomics, RNA sequencing and viral replication assays, we show that isolates of the Alpha (B.1.1.7) variant^3^ suppress innate immune responses in airway epithelial cells more effectively than first-wave isolates. We found that the Alpha variant has markedly increased subgenomic RNA and protein levels of the nucleocapsid protein (N), Orf9b and Orf6—all known innate immune antagonists. Expression of Orf9b alone suppressed the innate immune response through interaction with TOM70, a mitochondrial protein that is required for activation of the RNA-sensing adaptor MAVS. Moreover, the activity of Orf9b and its association with TOM70 was regulated by phosphorylation. We propose that more effective innate immune suppression, through enhanced expression of specific viral antagonist proteins, increases the likelihood of successful transmission of the Alpha variant, and may increase in vivo replication and duration of infection^4^. The importance of mutations outside the spike coding region in the adaptation of SARS-CoV-2 to humans is underscored by the observation that similar mutations exist in the N and Orf9b regulatory regions of the Delta and Omicron variants.

## Abstract (web summary)

The SARS-CoV-2 Alpha variant suppresses innate immune responses more effectively than isolates of first-wave SARS-CoV-2, and this is a result of mutations outside of the spike coding region that lead to upregulation of viral innate immune antagonists.

## Main

**Fig. 1:**
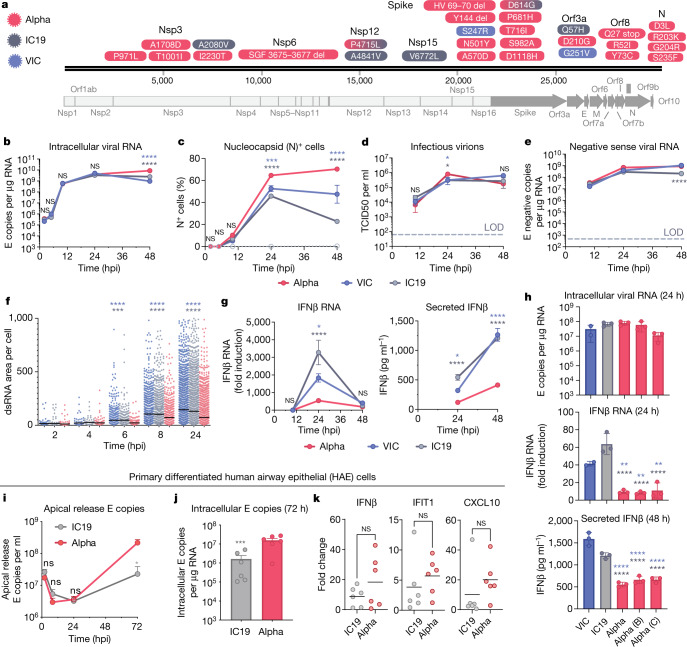
The SARS-CoV-2 Alpha variant antagonizes innate immune activation more efficiently than early-lineage isolates. **a**, Protein-coding changes in SARS-CoV2 Alpha (red), early-lineage IC19 (grey) and early-lineage VIC (blue) are indicated in comparison to the Wuhan-Hu-1 reference genome (MN908947). **b**–**e**, Viral replication after infection of Calu-3 cells with 5,000 E copies per cell. LOD, limit of detection. **b**, Intracellular viral RNA. **c**, Nucleocapsid (N)^+^ cells. **d**, Infectious virions (TCID50, 50% tissue culture infectious dose). **e**, Negative-sense viral RNA. **f**, Total area of dsRNA area per cell measured by single-cell immunofluorescence in Calu-3 cells infected with 2,000 E copies per cell. **g**, Expression and secretion of IFNβ by cells in **b**. **h**, Replication (intracellular viral RNA; 24 h) and IFNβ expression (24 h) and secretion (48 ) after infection of Calu-3 cells with 250 E copies per cell. **i**, **j**, Measurements of infection in primary differentiated HAE cells infected with 2,000 E copies per cell (**j**, 72 h). **k**, Expression of IFNβ and ISGs in cells from **j**. Mean ± s.e.m. of one of three representative experiments performed in triplicate. For **i**–**k**, *n* = 6, two independent donors. For **f**, one of two independent experiments with one data point per cell is shown. Two-way ANOVA (**b**–**e**) with Dunn’s multiple comparison test (**f**), one-way ANOVA with Tukey’s post-hoc test (**g**, **h**, **i**) or Wilcoxon matched-pairs signed rank test (**j**, **k**). Blue asterisks, Alpha versus VIC (blue lines and symbols); grey stars, Alpha versus IC19 (grey lines and symbols). *P < 0.05, **P < 0.01, ***P < 0.001, ****P < 0.0001; NS, not significant.

**Extended Data Fig. 1:**
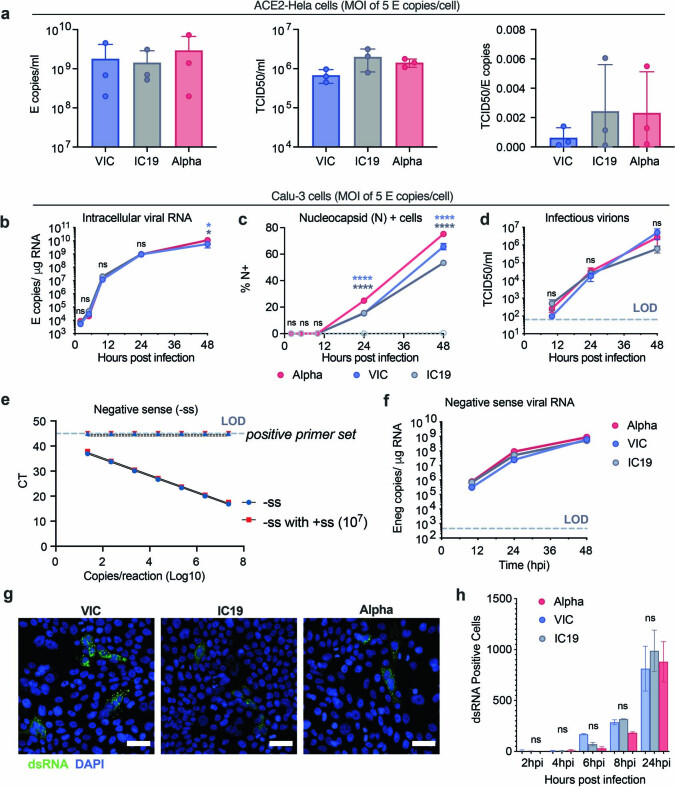
The SARS-CoV-2 Alpha variant replicates similarly to early-lineage isolates in Calu-3 cells. **a**, E copies/ml (left), TCID50/ml (centre) and infectious units per genome (TCID50/E copies) (right) were measured in viral stocks. **b**–**d**, Calu-3 cell infection with 5 E copies/cell. Viral replication (b), % infection (c), and infectious virion production (d) are shown. **e**, Quantification of E gene negative sense standard RNA in the presence and absence of 10^7^ positive sense E RNA copies. Positive sense E primer set run with negative sense standards, observed at the limit of detection. **f**. Negative sense E copies in cells from (b). **g**, **h**, dsRNA detection by single cell immunofluorescence in cells infected with 2,000 E copies/cell. Representative images at 24 hpi (g) and quantification of dsRNA-positive cells (h) are shown. Shown are mean ± s.e.m. of one of three representative experiments performed in triplicate. For (g) representative images from two independent experiments, quantified in (h), are shown. Scale bars are 50 μm. Two Way ANOVA (b,c,d,f) or One Way ANOVA with a Tukey post-hoc test were used. Blue stars indicate comparison between Alpha and VIC (blue lines and symbols), grey stars indicate comparison between Alpha and IC19 (grey lines and symbols). * (p < 0.05), ** (p < 0.01), *** (p < 0.001), **** (p < 0.0001). ns: non-significant. E: viral envelope gene. LOD, limit of detection.

Innate immunity exerts strong selective pressure during viral transmission^5–7^ and affects COVID-19 outcomes^8–10^. We hypothesized that the Alpha variant evolved enhanced innate immune escape through adaptations outside the spike proteins. Naturally permissive Calu-3 human lung epithelial cells infected with first-wave (early-lineage) SARS-CoV-2 induce a delayed innate response, which is driven by the activation of the RNA sensors RIG-I and MDA5 (ref. ^11^). Delayed responses, compared to rapid viral RNA replication, suggest effective early innate immune antagonism and evasion^12,13^. Here, we evaluated differences in replication and host responses to Alpha and first-wave isolates: B lineage BetaCoV/Australia/VIC01/2020 (VIC) and B.1.13 hCoV-19/England/IC19/2020 (IC19) (Fig. 1a). Input dose was normalized using viral genomic and subgenomic copies of envelope (E) RNA (quantitative PCR with reverse transcription; RT–qPCR). Dose normalization is critical because input viral genome levels correspond with innate immune activation at 24 hours post-infection (hpi) in Calu-3 cells^11^. Equalizing input genomes also allows assessment of infectivity per genome, which may vary between variants. We therefore confirmed that measurements of E copies and infectious virions in inocula correlate, and that the infectivity (infectious units per E copy), is comparable between Alpha and first-wave isolates, supporting our dosing approach (Extended Data Fig. 1a).

## Alpha shows reduced interferon induction

We found that the replication of Alpha and first-wave isolates was comparable at a high and a low multiplicity of infection (MOI), measuring intracellular E copies, N positivity and infectious virion production (Fig. 1b–d, Extended Data Fig. 1b–d). We observed a small but significant increase in N positivity after Alpha infection (Fig. 1c, Extended Data Fig. 1c), which we explain later. As double-stranded RNA (dsRNA) intermediates are important pathogen-associated molecular patterns (PAMPs) sensed by the cell^11,14^, we also confirmed equivalent negative-sense RNA synthesis for Alpha and first-wave isolates (Fig. 1e, Extended Data Fig. 1f), using strand-specific RT–qPCR (Extended Data Fig. 1e). All isolates reached comparable levels of dsRNA-positive cells from 8 hpi (Extended Data Fig. 1g, h). However, Alpha isolates exhibited a reduction in the total area of dsRNA per cell from 6 hpi, despite replication being otherwise comparable (Fig. 1f). One possibility is that increased levels of the Alpha N protein (Fig. 1c, Extended Data Fig. 1c, Fig. 3) contribute to innate immune evasion by sequestering dsRNA, causing epitope masking. Alternatively, Alpha may induce less endogenous dsRNA production from the expression of transposable elements that can contribute PAMPs to innate immune sensing^15–17^.

**Extended Data Fig. 2:**
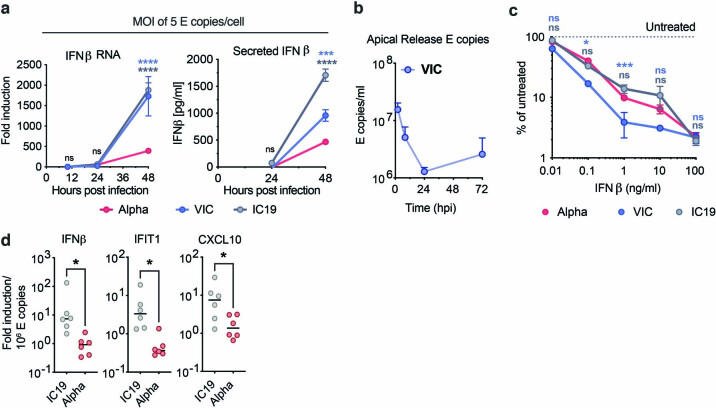
The SARS-CoV-2 Alpha variant antagonizes innate immune activation more efficiently than early-lineage isolates. **a**, IFNβ gene expression (left) and protein secretion (right) from cells in Extended Data Fig 1b. **b**, HAE cells were infected with 2,000 E copies/cell of VIC. E copies were measured in apical washes of infected cultures. **c**, Calu-3 infection at 2,000 E copies/cell after 8h pre-treatment with IFNβ. Infection levels are shown normalized to untreated controls at 24 hpi. **d**, IFNβ and ISGs expression in HAE cells infected with 2,000 E copies/cell of IC19 or Alpha variant normalized to intracellular E copies for each sample. Shown are mean ± s.e.m. of one of three representative experiments performed in triplicate. For d, n = 6, two independent donors. Two Way ANOVA (a,c) or One Way ANOVA (d) with Wilcoxon matched-pairs signed rank test were used. Blue stars indicate comparison between Alpha and VIC (blue lines and symbols), grey stars indicate comparison between Alpha and IC19 (grey lines and symbols). * (p < 0.05), ** (p < 0.01), *** (p < 0.001), **** (p < 0.0001). ns: non-significant. E: viral envelope gene.

Identical levels of replication of each isolate enabled direct comparison of innate immune responses without confounding differences in the amount of virus. We found that Alpha infection led to lower expression and secretion of interferon-β (IFNβ) (Fig. 1g, Extended Data Fig. 2a), a result that was confirmed with three independent Alpha isolates (Fig. 1h). Differences in innate immune activation between variants did not translate to differences in viral replication in Calu-3 cells (Fig. 1). We therefore compared replication and innate immune activation in primary human airway epithelial (HAE) cells differentiated at an air–liquid interface. Alpha showed enhanced replication in HAE cells (Fig. 1i, j); the replication of VIC was particularly limited (Extended Data Fig. 2b), probably owing to the absence of the D614G mutation in the spike protein, which confers a replication advantage in HAE cells and animal models^18–20^.

Thus, we compared innate replication and immune activation between Alpha and IC19 and found that innate activation was similar at 72 hpi (Fig. 1k), despite substantially enhanced Alpha replication (Fig. 1i, j). Viral replication was not increased beyond input levels at early time points (24 hpi; Fig. 1i), therefore interferon-stimulated genes (ISGs) were not induced (data not shown). However, when innate immune activation was normalized for viral replication at 72 hpi, with the caveat that E copies may not fully represent the amount of viral dsRNA PAMPs, we found that Alpha induced less expression of IFNβ and ISGs than did IC19 per E copy (Extended Data Fig. 2d). This is consistent both with enhanced innate immune antagonism by Alpha and with similar innate immune activation in Fig. 1k, as Alpha replicates more efficiently in primary HAE cells.

As IFN sensitivity correlates with the transmission of other pandemic viruses^5,6^, we measured IFNβ sensitivity. Alpha was consistently less sensitive to IFNβ over a wide range of doses compared to VIC (Extended Data Fig. 2c). Notably, IC19 showed a similar reduction in IFNβ sensitivity to Alpha (Extended Data Fig. 2c), perhaps owing to the D614G change in the spike protein, which is shared between IC19 and Alpha; this mutation is associated with IFN resistance and enhanced entry efficiency^18,21–23^. Thus Alpha not only induces less IFNβ (Fig. 1g, h, k, Extended Data Fig. 2a), but is also less sensitive to inhibition.

## Enhanced innate antagonism by Alpha

**Fig. 2:**
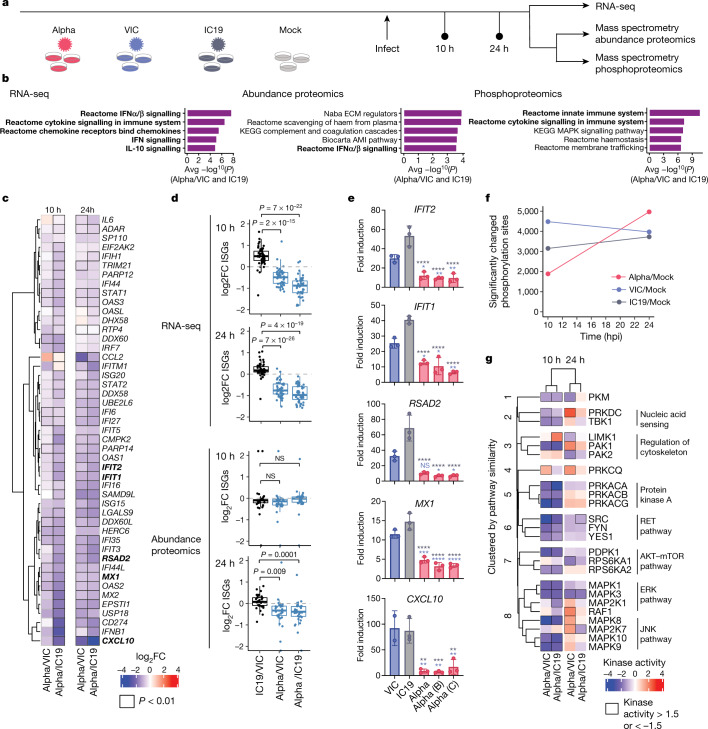
Global RNA-seq and proteomics reveal innate immune suppression by Alpha. **a**, Schematic of the experimental workflow. Calu-3 cells were infected with 5,000 E copies per cell of SARS-CoV-2 Alpha (red), early-lineage VIC (blue) or early-lineage IC19 (grey) or mock-infected (biological triplicates were performed for each time point). Phosphoproteomics and abundance proteomics analysis using a data-independent acquisition (DIA) and total RNA-seq were performed at 10 and 24 h. **b**, Unbiased pathway enrichment analysis. The −log_10_(*P*) values were averaged for enrichments using Alpha/VIC and Alpha/IC19 at 10 and 24 hpi to rank terms. The top five terms are shown. Innate immune system terms are shown in bold. ECM, extracellular matrix; AMI, acute myocardial infarction. **c**, Heat map depicting the log_2_-transformed fold change (log_2_FC; colour) of ISGs^25^ (by RNA-seq) comparing Alpha to VIC or IC19. Black outlines indicate *P* < 0.01. **d**, Box plots show log_2_FC of ISGs between Alpha/VIC, Alpha/IC19 or IC19/VIC. Dots indicate different ISGs. Boxes indicate median (middle line) and interquartile range (upper and lower lines). Blue indicates comparisons with Alpha; black indicates comparisons between early-lineage viruses (IC19 and VIC). **e**, RT–qPCR analysis of bolded ISGs from **c** in cells infected with 2,000 E copies per cell. Mean ± s.e.m. **f**, Number of phosphorylation sites significantly dysregulated for Alpha, VIC or IC19 versus mock at an absolute log_2_FC > 1 and adjusted *P* < 0.05. **g**, Kinase activities for the top enriched terms for the phosphoproteomics dataset ‘Reactome innate immune system’ (**b**, right). Two-tailed student’s *t*-test (**d**) or two-way ANOVA with Tukey’s multiple comparisons post-hoc test (**e**). Blue asterisks, Alpha versus VIC (blue bars); grey stars, Alpha versus IC19 (grey bars). *P < 0.05, **P < 0.01, ***P < 0.001, ****P < 0.0001, or exact *P* value (**d**); NS, not significant.

**Extended Data Fig. 3:**
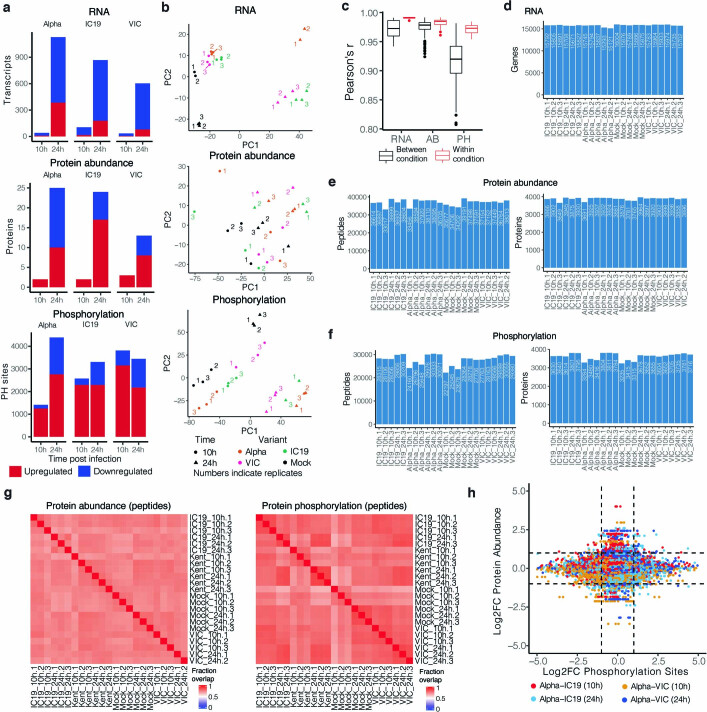
Omics data quality control and pathway enrichments. **a**, Significantly changing genes for RNA, proteins for protein abundance, and phosphorylation sites for phosphoproteomics data. Significance was defined as abs(log2FC)>1 and adjusted p-value < 0.05. Red depicts positive log2 fold changes whereas blue depicts negative log2 fold changes. **b**, Principal components analysis (PCA) on normalized RNA transcripts per million (TPM), protein intensities, or phosphorylation site intensities. Non-finite values were removed and detections (transcripts, proteins, or phosphorylation sites) not shared (non-finite) between all conditions were discarded prior to analysis. Coloured numbers indicate biological replicates. **c**, Pairwise Pearson’s correlation between RNA, protein, or phosphorylation site abundance among replicates within the same condition (red) or between distinct conditions (black). **d**, Number of genes expressed above baseline in RNA-seq dataset per replicate. **e**, Number of peptides and proteins detected per replicate in the abundance proteomics dataset. **f**, Number of phosphorylated peptides and corresponding proteins from the phosphoproteomics dataset. **g**, Fraction of peptides from protein abundance (left) or phosphoproteomics (right; phosphorylated peptides) that overlap between two replicates. **h**, Correlation between Log2 fold-change (log2FC) phosphorylation sites and log2FC abundance of the corresponding protein. Dots are coloured according to the comparison between conditions.

To compare global host responses to SARS-CoV-2 variants, we performed mass spectrometry protein abundance and phosphorylation profiling and total RNA sequencing (RNA-seq) in Calu-3 cells at 10 and 24 hpi (Fig. 2a, Supplementary Table 1). We observed infection-driven changes in RNA abundance and protein phosphorylation, with fewer differences in protein abundance (Extended Data Fig. 3a). We also observed poor correlation between protein phosphorylation and protein or mRNA abundance, suggesting that phosphorylation is driven independently from changes in protein abundance (Extended Data Fig. 3h).

**Extended Data Fig. 4:**
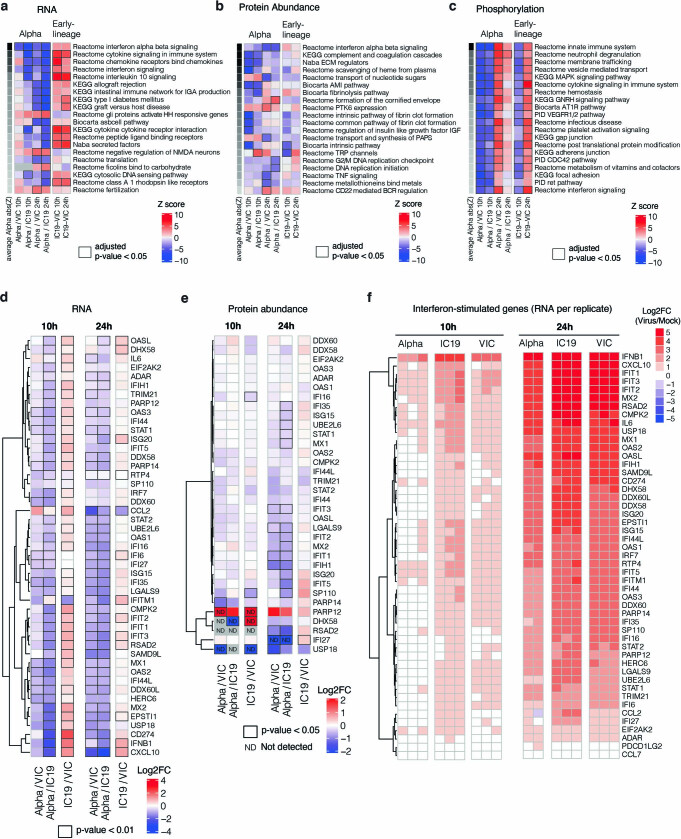
Omics data highlight the recruitment of innate immune signalling. **a**, Gene set enrichment analysis based on log2FC method using RNA dataset (as in Fig. 2b). Ranking is based on the average of the absolute value z-scores across the indicated contrasts involving Alpha (per row). Black borders indicate an adjusted p-value < 0.05. **b**, Same as in a, but for abundance proteomics dataset. **c**, Same as in a, but for phosphoproteomics dataset. If a protein possessed multiple phosphorylation sites, the maximum absolute value log2FC was used as the representative value for the protein. Finite values (non-infinite) were prioritized over quantitative values. **d**, Expression of interferon-stimulated genes from Lui et al (2018)^25^ (see Methods) using the RNA-seq dataset. Significant fold changes with an adjusted p-value < 0.05 are indicated with black borders. **e**, Same as in (a) using the abundance proteomics dataset. N.D. indicates proteins either not detected in one condition (thus, Inf or -Inf) or not detected in both conditions. **f**, RNA expression per biological replicate of interferon-stimulated genes (ISGs) for each virus versus mock.

**Extended Data Fig. 5:**
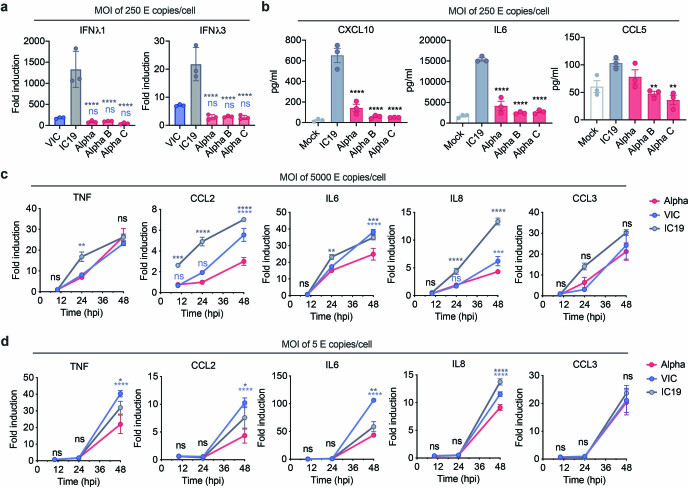
Infection with the SARS-CoV-2 Alpha variant results in lower IFN III and pro-inflammatory responses than first wave isolates. **a**, Calu-3 cells were infected with 250 E copies/cell and *IFNL1* and *IFNL3* expression measured at 24 hpi. **b**, Secretion of CXCL10, IL6 and CCL5 by infected cells at 48 hpi. **c**, **d**, Calu-3 cells were infected with (c) 5,000 E copies/cell or (d) 5 E copies/cell. Expression of *TNF*, *CCL2*, *IL6*, *IL8* and *CCL3* were measured. Data shown are mean ± s.e.m. of one of three representative experiments performed in triplicate. One Way ANOVA with a Tukey post-comparison test (a, b) or two Way ANOVA (c,d) were used. Blue stars indicate comparison between Alpha and VIC (blue lines and symbols), grey stars indicate comparison between Alpha and IC19 (grey lines and symbols). * (p < 0.05), ** (p < 0.01), *** (p < 0.001), **** (p < 0.0001). ns: non-significant. E: viral envelope gene.

Gene set enrichment analysis^24^ (GSEA) comparing Alpha to first-wave isolates highlighted pathways that relate to the innate immune system among the top five terms for RNA, protein abundance and phosphorylation (Fig. 2b, Extended Data Fig. 4a–c, Supplementary Table 2). The highest-scoring terms were related to IFNα, IFNβ, cytokine and chemokine signalling, and were most enriched for the RNA and protein phosphorylation datasets (Fig. 2b). In addition to lower production of IFNβ (Fig. 1g, h, Extended Data Fig. 2a, d), infection with Alpha resulted in reduced expression of ISGs in RNA-seq data (10 and 24 hpi) and protein abundance data (24 hpi) using an ISG set^25^ (Methods, Supplementary Table 3, Fig. 2c, d, Extended Data Fig. 4d–f). For a subset of genes (*CXCL10*, *IFIT2*, *MX1*, *IFIT1* and *RSAD2*) (Fig. 2e), as well as type III IFNλ1 and IFNλ3 (Extended Data Fig. 5a), we confirmed reduced induction by multiple Alpha isolates (RT–qPCR).

**Fig. 3:**
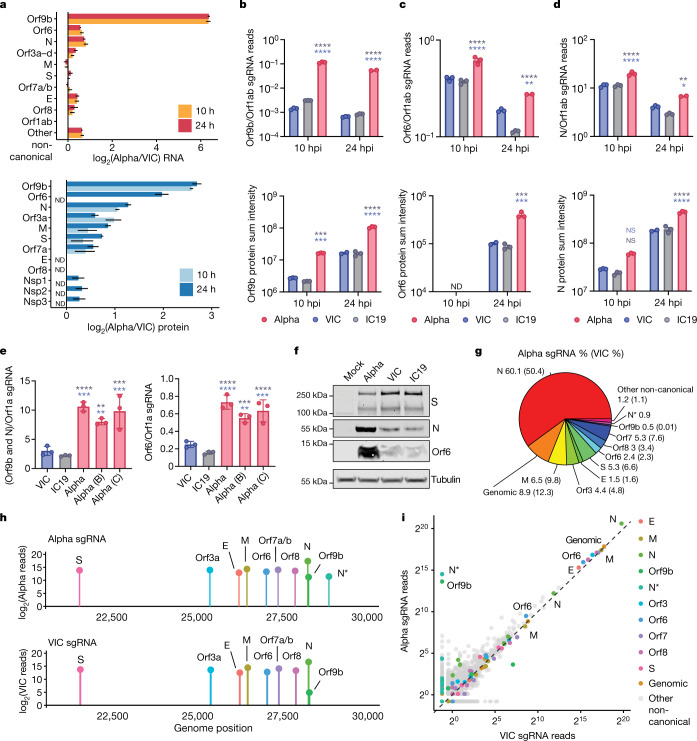
The SARS-CoV-2 Alpha variant upregulates innate immune antagonists at the subgenomic RNA and protein level. **a**, Top, the log_2_ ratio of Alpha to VIC sgRNA normalized to total genomic RNA per time point and virus (from RNA-seq). Bottom, the log_2_ ratio of summed peptide intensities per viral protein comparing Alpha to VIC (from proteomics analysis) (*n* = 3). Orf3a–d refers to Orf3a, Orf3b, Orf3c and Orf3d. S, spike protein; E, envelope protein; M, membrane protein. ND, not detected. **b**–**d**, Quantification of Orf9b (**b**), Orf6 (**c**) and N (**d**) sgRNA from the RNA-seq dataset (top) and summed peptides per viral protein (bottom). **e**, Quantification of Orf9b and N (left) or Orf6 (right) sgRNA abundance by RT–qPCR (24 hpi). **f**, Representative western blot of Orf6, N and S expression in infected Calu-3 cells (2,000 E copies per cell) at 24 hpi (*n* = 3). **g**, Pie chart depicting the proportion (shown as percentages) of total sgRNA mapping to each viral sgRNA for Alpha at 24 hpi. VIC percentages in parentheses. **h**, sgRNA log_2_-normalized counts (dot height) projected onto their identified start sites on the SARS-CoV-2 genome (24 hpi). Canonical and two non-canonical sgRNAs (Orf9b and N*) are depicted. **i**, Scatter plot of sgRNA abundance in Alpha or VIC at 24 hpi. Grey dots indicate other non-canonical sgRNAs containing a leader sequence but no clear start codon. Mean ± s.e.m. (**a**–**e**). Two-way ANOVA with Tukey’s multiple comparisons post-hoc test (**c**–**e**). *P < 0.05, **P < 0.01, ***P < 0.001, ****P < 0.0001; NS, not significant.

**Extended Data Fig. 6:**
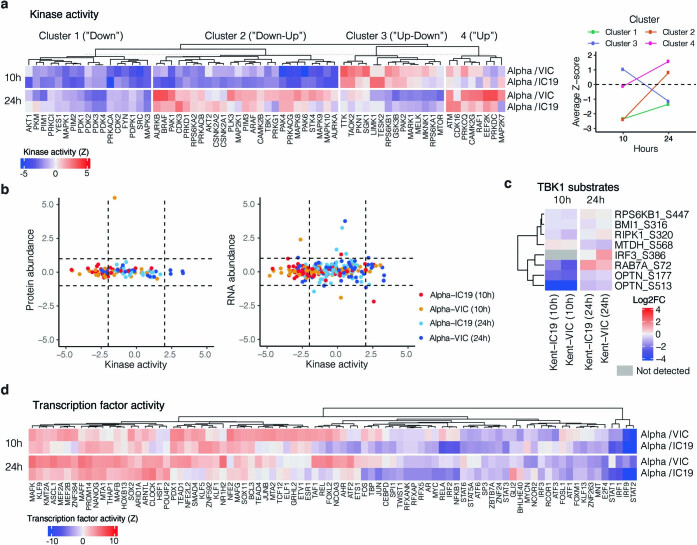
Kinase and transcription factor activity analysis. **a**, Full kinase activity analysis of indicated contrasts with z-score>2. Kinases were separated using k-means clustering, which naturally reveals groups depicting kinases downregulated for the entire time course (“Down”), downregulated early and upregulated late (“Down-Up”), upregulated early and downregulated late (“Up-Down”), or upregulated or constant throughout the time course (“Up”). Panel on the right depicts the average Z-score for each distinct cluster per time point, collapsing across Alpha/VIC and Alpha/IC19 comparisons. **b**, Correlation between the calculated kinase activity Z-score and protein (left) or RNA (right) abundance log2FC for kinases with estimated activities in our dataset. Vertical dashed lines indicate kinase activity of ±2, horizontal dashed lines indicate protein log2FC of ±1. Colours represent comparisons between viruses and time points as indicated. **c**, Detected substrates known to be phosphorylated by TBK1. Log2FC of each phosphorylation site is depicted. Those not detected are indicated in grey. **d**, Transcription factor (TF) activities were estimated from the RNA-seq dataset using known TF-target gene interactions. Included are TFs with a NES>2.5. TF are clustered using ward hierarchical clustering based on similar activity patterns across time.

We observed lower overall changes in protein phosphorylation early in infection for Alpha (Fig. 2f). Accordingly, GSEA revealed that pathways with reduced phosphorylation at 10 hpi—that is, decreased activation—are related to innate immune responses (Extended Data Fig. 4c), consistent with enhanced antagonism by Alpha. Notably, this was reversed at 24 hpi as Alpha caused enhanced phosphorylation later in infection (Extended Data Fig. 4c). This led us to investigate the differential regulation of kinase signalling cascades, especially with respect to innate immune signalling. We used the phosphoproteomics data to estimate kinase activities for 191 kinases on the basis of regulation of their known substrates^26,27^ (Supplementary Table 4), and grouped kinases according to their temporal dynamics (Extended Data Fig. 6a). Of note, we did not observe any correlation between kinase activity and abundance in protein and RNA datasets (Extended Data Fig. 6b), suggesting that changes in kinase activity are not driven by corresponding changes in kinase abundance. We identified 24 kinases from the top enriched term (‘Reactome innate immune system’; Fig. 2b), which we clustered by similar pathway membership (Fig. 2g, Methods). At 10 hpi, we observed decreased activity of TBK1, as well as protein kinase A, PRKDC, RET, AKT–mTOR, ERK and JNK pathways. Given the central role of TBK1 in nucleic acid sensing, we evaluated known TBK1 substrates in greater detail to support the kinase analysis (Fig. 2g), and confirmed the lower levels of phosphorylation of known TBK1 substrates, including OPTN (ref. ^28^) and Ser72 in RAB7A (ref. ^29^), for Alpha compared to first-wave isolates at 10 hpi (Extended Data Fig. 6c). At 24 hpi, the activity of TBK1 and PRKDC kinases, as well as that of JNK, ERK and PKA pathway kinases, was increased for Alpha compared to VIC (Fig. 2g), consistent with the increased phosphorylation in innate-immune-system-enriched pathway terms (Extended Data Fig. 4c). Persistently lower induction of IFN by Alpha at 24 and 48 hpi (Figs. 1, 2, Extended Data Fig. 1), despite higher activation of TBK1 at 24 hpi, suggests antagonism downstream of TBK1; for example, by increased expression of SARS-CoV-2 Orf6 (Fig. 3), which suppresses the nuclear transport of inflammatory transcription factors^13^. Concordantly, pro-inflammatory mRNA induction (*IL6*, *IL8*, *CCL2* and *TNF*) and cytokine release (CXCL10, IL6 and CCL5) were significantly lower after infection with Alpha, compared to first-wave isolates (Extended Data Fig. 5b–d). This is consistent with a sustained reduction in cellular activation driven by inhibition of pathways upstream and downstream of TBK1 by Alpha. We did not observe differences in CCL3 induction, suggesting that not all inflammatory pathways are differentially regulated between viruses (Extended Data Fig. 5c, d). Thus, Alpha-enhanced innate immune antagonism, as judged by decreased protein phosphorylation, is only observed at early time points after infection, suggesting a delayed activation of signalling pathways involved in viral recognition compared to early-lineage viruses.

## Higher expression of innate antagonists by Alpha

**Extended Data Fig. 7:**
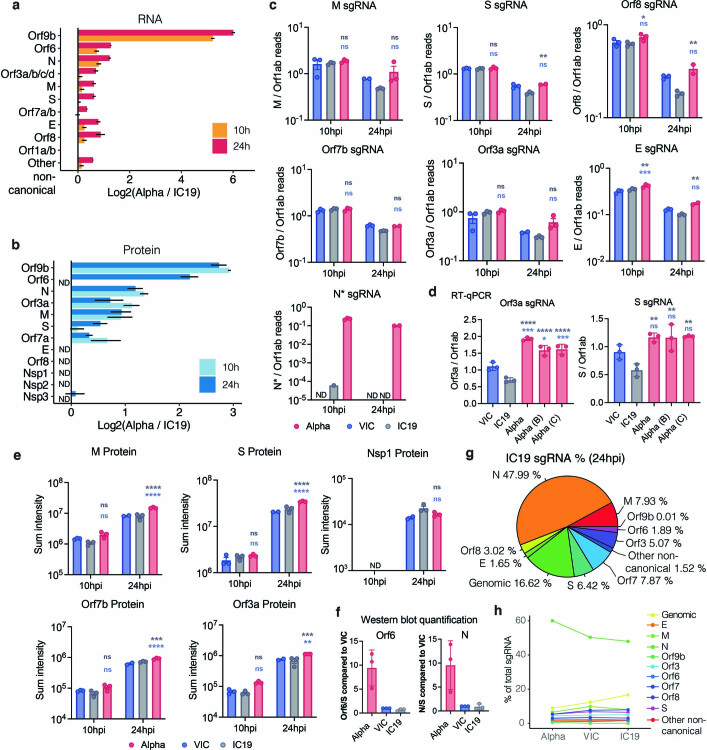
Expression of viral RNA and protein for SARS-CoV-2 variants. **a**, Log2 ratio of Alpha to IC19 subgenomic RNA (sgRNA) abundance as determined from the RNA-seq dataset. **b**, Log2 ratio of Alpha to IC19 viral proteins. Peptide intensities are summed per viral protein (n = 3). **c**, Quantification of sgRNAs for M, S, Orf8, Orf7a, Orf3a, E and N* from the RNA-seq dataset. Counts are normalized to genomic RNA abundance at each time point and virus. **d**, Quantification of Orf3a (left) or S (right) sgRNA abundance via RT–qPCR. **e**, Summed peptides per viral protein for M, S, Nsp1, Orf7b, and Orf3b. **f**, Western blot quantification of Orf6 and N protein in infected cells at 24 hpi (n = 3). **g**, Pie chart depicting proportion of total sgRNA mapping to each viral sgRNA for IC19. **h**, Mean ± s.e.m. are shown. Comparison of percentages of total sgRNA mapping to each viral sgRNA across Alpha, VIC, and IC19. * (p < 0.05), ** (p < 0.01), *** (p < 0.001), **** (p < 0.0001). ns: non-significant, ND, not detected.

We next examined the viral RNA-seq and proteomic data, seeking to understand the differences between Alpha and first-wave isolates that underlie the contrasting host responses (Fig. 3a, Extended Data Fig. 7a, b, Supplementary Tables 6, 7). As RNA replication, measured by the levels of genomic and subgenomic (sgRNA) E, was similar between variants (Fig. 1, Extended Data Fig. 1), we determined the levels of each sgRNA by selecting transcripts with the 5′ leader sequence, derived from the 5′ genomic RNA during sgRNA synthesis (Fig. 3a, Extended Data Fig. 7). We observed similar levels of Nsp1, Nsp2 and Nsp3 proteins (Orf1ab) translated from genomic RNA (Fig. 3a), which is again consistent with comparable levels of infection, and thus enables effective comparisons of transcription and protein expression between variants.

**Extended Data Fig. 8:**
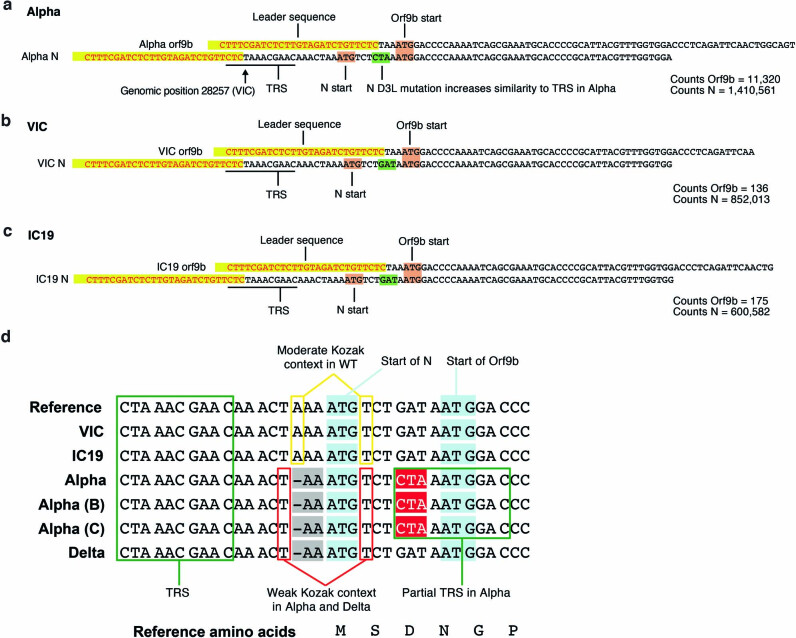
Examples of leader-containing reads for Orf9b and N from the RNA-seq dataset. **a**–**c**, Representative sequence for Orf9b (top) and N (bottom) sgRNA from Alpha (a), VIC (b) and IC19 (c). Leader sequences to identify sgRNAs are highlighted in yellow. The following sequence is used to differentiate Orf9b versus N sgRNAs. Orf9b and N start codons shown in maroon. The site of the N-protein D3L mutation is indicated in green, resulting in increased similarity to the transcriptional regulatory sequence (TRS) for Alpha. Read counts of Orf9b and N are indicated to the right. Counts are normalized to mean genomic reads per replicate.

Notably, we found a large increase in the innate immune antagonist Orf9b (97-amino-acid version^30^, encoded by an alternative reading frame within N) in Alpha compared to first-wave isolates (Fig. 3a, b Extended Data Fig. 7b), with a corresponding increase in Orf9b sgRNA^31^ (an increase of more than 80-fold for Alpha sgRNA compared to VIC, and 64.5-fold for Alpha compared to IC19, at 24 hpi; Fig. 3a, b, Extended Data Fig. 7a). The increase in Orf9b transcription in Alpha is likely to be influenced by nucleotide changes 28,280 GAT>CTA (conferring the D3L substitution in the N protein), which introduces an enhanced transcriptional regulatory sequence (TRS) upstream of Orf9b^31^ (Extended Data Fig. 8a–c). However, the overall amount of Alpha Orf9b sgRNA remains low (Fig. 3g). Thus, it is possible that increased expression of the Orf9b protein also derives from enhanced leaky scanning of the N sgRNA owing to a single-nucleotide deletion that weakens the Alpha N Kozak translation initiation context (position 28,271 in VIC and IC19; Fig. 6). The three-nucleotide mutation leading to N(D3L) also modifies the Alpha Orf9b Kozak context, which could influence Orf9b translation efficiency^32^. We predict a complex interplay between mutations that results in the enhancement of both Orf9b and N expression.

We also found that Alpha had a significant increase in sgRNA and protein expression (24 hpi) for a second innate immune regulator, Orf6^12,13^ (Fig. 3a, c, Extended Data Fig. 7a, Supplementary Table 6). The specific mutations that influence Orf6 expression remain unclear. In addition, we detected increased sgRNA and protein levels in Alpha of N, a third innate immune regulator^33^ (Fig. 3a, d). This is consistent with the increase in N-positive cells measured during Calu-3 infection (Fig. 1c, Extended Data Fig. 1c). We also observed enhancement of Orf3a, membrane (M) and Orf7b proteins at 24 hpi for Alpha, with only very modest changes observed at the RNA level (Fig. 3a, Extended Data Fig. 7a, c, d). We confirmed the upregulation of Alpha Orf9b, N and Orf6 sgRNA using RT–qPCR (Fig. 3e) and the increased expression of Alpha Orf6 and N proteins by immunoblot (Fig. 3f). These findings are consistent with the reported enhanced expression of Alpha Orf9b, Orf6 and N sgRNA in clinical samples^31^. The proportion of each sgRNA of the total sgRNA reads is summarized for each variant in Fig. 3g and Extended Data Fig. 7g. Of note, we observed an additional sgRNA in Alpha, called N* (ref. ^31^), with an in-frame start codon at N M210 encoding the C terminus of the N protein (Fig. 3h, Supplementary Table 7). N* synthesis is likely driven by the triple nucleotide mutations (encoding the R203K/G204R substitutions in the Alpha N protein) just upstream of the N* start codon, which create a new TRS for N* transcription, as previously suggested^31^. Accordingly, we did not detect N* sgRNA in VIC or IC19 above background levels, while it accounted for 0.9% of the total sgRNA in Alpha (Fig. 3g) . Indeed, measurements of sgRNA abundance were consistent with Orf9b and N* being the most differentially expressed sgRNA between Alpha and first-wave isolates (Fig. 3i, Extended Data Fig. 7c). We note that Alpha sgRNA synthesis is not universally increased (Fig. 3a), because M and spike sgRNAs are not enhanced.

## Phosphorylation regulates Orf9b activity

**Fig. 4:**
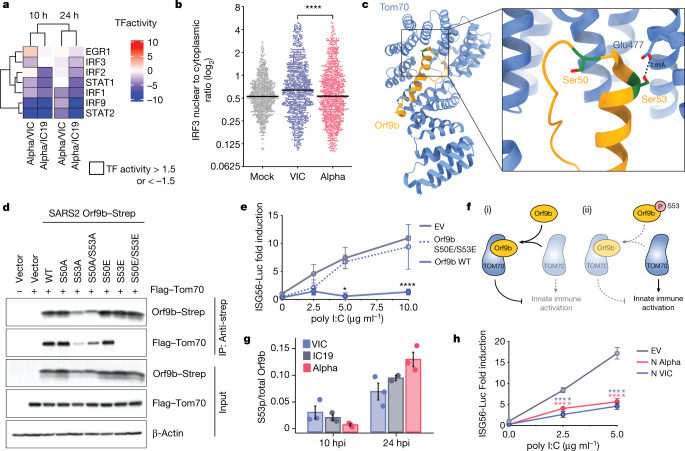
Orf9b binds TOM70 and antagonizes innate immune activation downstream of RNA sensing. **a**, Transcription factor (TF) activities in the five top enriched terms for the RNA-seq dataset (Fig. 2b, left); rows clustered hierarchically based on activity magnitude. Black outlines show activities >1.5 or  < −1.5. **b**, IRF3 nuclear to cytoplasmic ratio measured by single-cell immunofluorescence at 24 h in cells infected at 2,000 E copies per cell; 1,000 randomly sampled cells per condition (cut-off of 0.1> = <5). **c**, Cryo-electron microscopy of SARS-CoV-2 Orf9b (yellow) in complex with TOM70 (blue) (Protein Data bank (PDB) code: 7KDT)^40^. Serine residues (Ser50 and Ser53) in Orf9b in the TOM70-binding site are shown in red. **d**, Co-immunoprecipitation of Orf9b wild type (WT) or point mutants with TOM70 in HEK293T cells. **e**, ISG56-reporter activation by poly I:C in the presence of Orf9b WT, S50E/S53E or empty vector (EV) in HEK293T cells. **f**, Schematic of proposed innate immune antagonism by Orf9b. (i) When S53 is unphosphorylated, Orf9b binds TOM70 to inhibit innate immune signalling. (ii) When S53 is phosphorylated, Orf9b can no longer interact or antagonize innate immune activation. **g**, Ratio between the intensity of Orf9b peptide phosphorylated on Ser53 (S53p) and total Orf9b (as calculated in Fig. 3b, bottom) from phospho- and abundance proteomics of Calu-3 cells (Fig. 2). **h**, ISG56-reporter activation by poly:IC in the presence of N (VIC), N (Alpha) or EV in HEK293T cells. Mean ± s.e.m. Mann–Whitney test (**b**) or two-way ANOVA with Tukey’s post-hoc test (**e**, **h**). For **e**, Orf9b WT versus Orf9b(S50E/S53E). For **h**, blue stars: VIC versus EV; red stars, Alpha versus EV. *P* < 0.05, **P < 0.01, ***P < 0.001, ****P < 0.0001.

To further understand differences in host responses to Alpha, we used the RNA-seq dataset to estimate transcription factor activities by mapping target genes to corresponding transcriptional regulators (Extended Data Fig. 6d, Supplementary Table 5). We extracted significantly regulated transcription factors within the top five most enriched terms from the unbiased RNA-seq pathway enrichment analysis (Fig. 2b). This revealed that IRF and STAT transcription factor families are significantly less activated by Alpha than by first-wave viruses (Fig. 4a). Consistently, measuring IRF3 nuclear translocation by single-cell immunofluorescence showed reduced activation of IRF3 after infection with Alpha compared to infection with VIC (Fig. 4b). STAT1, STAT2 and IRF9 lie downstream of the type I IFN receptor, and potent inhibition by Alpha is consistent with increased levels of Orf6, which is known to inhibit the nuclear translocation of STAT1 and IRF3 (refs. ^12,13^).

Decreased activation of TBK1 by Alpha (Fig. 2g) also suggests antagonism upstream of IRF3 by additional mechanisms. The N protein is reported to antagonize the activation of RNA sensors^33^. Alpha N has four coding changes as compared to first-wave viruses (Fig. 1a). However, the antagonism of poly I:C activation of an *ISG56*-luciferase reporter by Alpha N was comparable to antagonism by the N protein of first-wave viruses, suggesting that these coding changes do not enhance the potency of innate antagonism for Alpha N (Fig. 4h). Nonetheless, increased levels of Alpha N during infection may facilitate innate antagonism and evasion through enhanced sequestration of viral and host-derived PAMPs^34^ (Fig. 1f).

**Extended Data Fig. 9:**
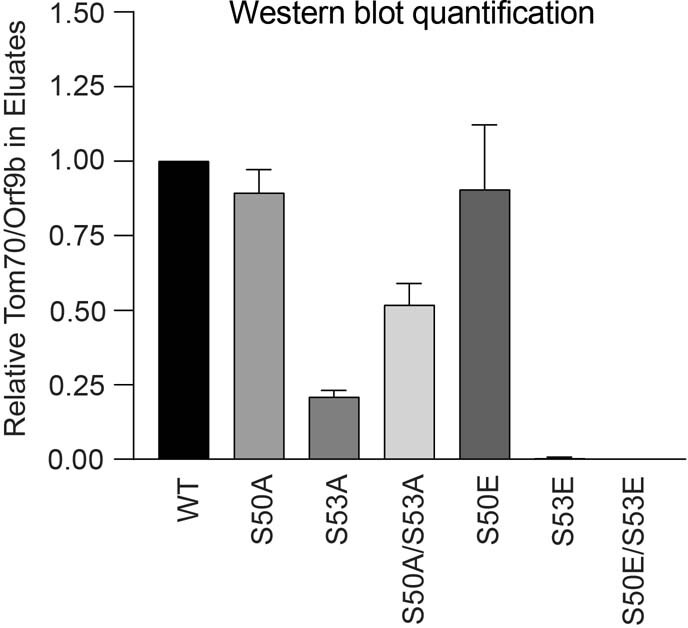
Western blot densitometry quantification for Orf9b immunoprecipitation with TOM70. Densitometry quantification of two western blot experimental repeats of Orf9b immunoprecipitation with TOM70 (as in Fig. 4d).

We have previously reported that SARS-CoV-2 Orf9b, which is expressed to significantly higher levels by Alpha (Fig. 3), interacts with human TOM70^35^, a mitochondrial import receptor that is required for the MAVS activation of TBK1 and IRF3 and subsequent RNA-sensing responses^36,37^. We previously found that two serine residues buried within the Orf9b–TOM70-binding pocket, Orf9b Ser50 and Ser53, are phosphorylated during SARS-CoV-2 infection^38–40^ (Fig. 4c). Here we discovered that mutating Ser53 alone or both Ser50 and Ser53 in Orf9b to the phosphomimetic glutamic acid residue disrupted the co-immunoprecipitation of Orf9b and TOM70 (Fig 4d) and abolished Orf9b antagonism of *ISG56-*luciferase reporter gene activation by poly I:C (Fig. 4e), presumably by preventing interaction with TOM70 (Fig. 4c). In addition, although the S53A mutation compromised protein stability (evidenced by immunoblot density, Extended Data Fig. 9), it confirmed the contribution of Ser53 to TOM70 binding, because S53A immunoprecipitated less TOM70 when normalized for Orf9b protein levels (Fig. 4d, Extended Data Fig. 9). Although it is unclear which kinases are responsible for Orf9b phosphorylation, our data are consistent with Orf9b suppressing signalling downstream of MAVS, by targeting TOM70, and also the regulation of Orf9b by host-mediated phosphorylation (Fig. 4f). Notably, we detected lower levels of Alpha Orf9b Ser53 phosphorylation at 10 hpi, but higher levels at 24 hpi, compared to first-wave isolates (Fig. 4g). This suggests that not only does Alpha express more Orf9b early in infection, but it may also be regulated more effectively by unknown host kinases to manipulate host innate immunity, consistent with enhanced host adaptation by Alpha.

## Discussion

**Fig. 5:**
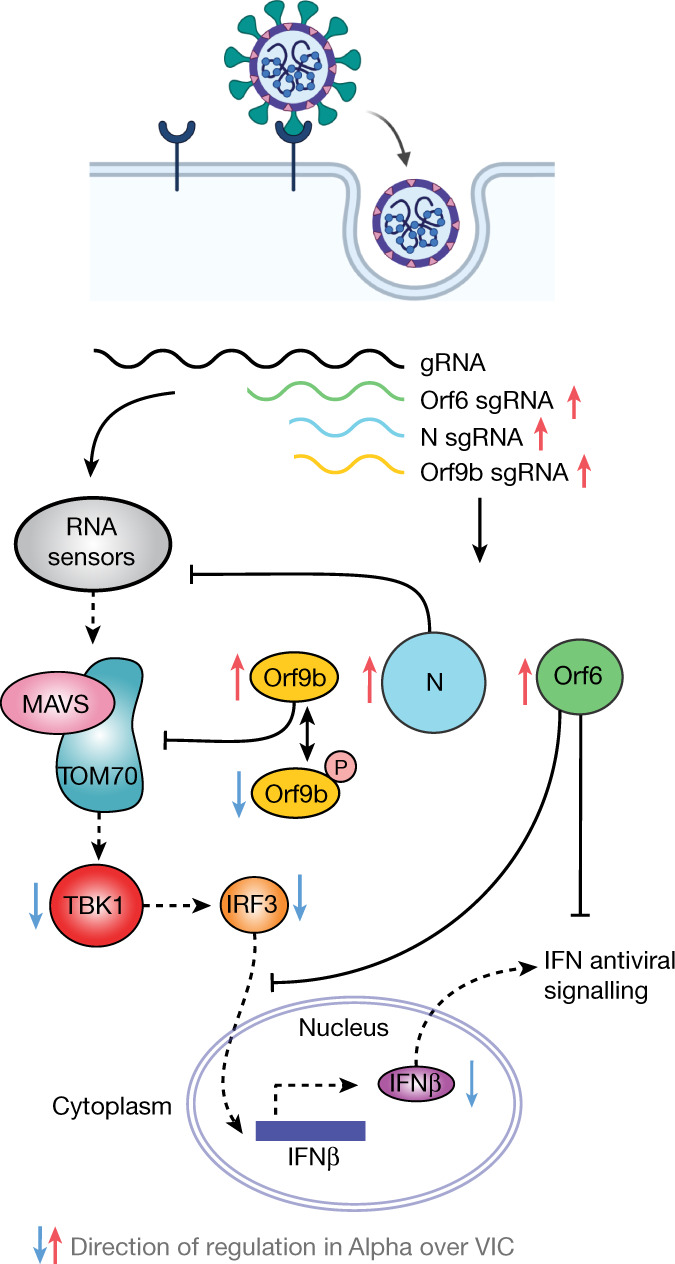
Antagonism of innate immune activation by Alpha. SARS-CoV-2 Alpha has evolved more effective innate immune antagonisms. First-wave isolates activate a delayed innate response in airway epithelial cells relative to rapid viral replication, indicative of viral innate immune antagonism early in infection. The known innate immune antagonists Orf9b, Orf6 and N act at different levels to inhibit RNA sensing. Orf6 inhibits IRF3 and STAT1 nuclear translocation^12,13^; N prevents activation of the RNA sensor RIG-I^33^; and Orf9b inhibits RNA sensing through interaction with TOM70, regulated by phosphorylation. Alpha has evolved to produce more sgRNA for these key innate immune antagonists, which leads to increased protein levels and enhanced innate immune antagonism as compared to first-wave isolates. gRNA, genomic RNA.

Our data reveal that changes outside the spike protein—including noncoding changes—are important in SARS-CoV-2 adaptation through influencing sgRNA and protein expression. For Alpha, we discovered an upregulation of key viral innate antagonists, Orf9b, Orf6 and N, leading to enhanced innate immune evasion (Fig. 5). We propose that in vivo, enhanced innate immune antagonism by Alpha contributes to its transmission advantage, by enhancing replication through reducing or delaying early host innate responses, which otherwise protect airway cells from infection and limit viral dissemination. This is also consistent with reports of prolonged viral shedding of Alpha^41,42^, suggesting less effective control of replication. Enhanced innate evasion has also been linked to transmission of HIV^5,6^.

**Fig. 6:**
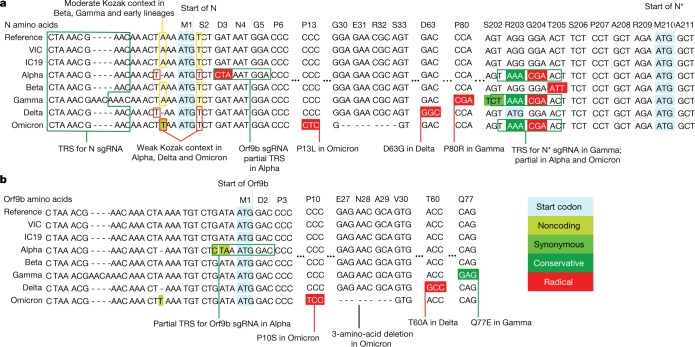
VOCs present similar nucleotide mutations in N and Orf9b. **a**, **b**, Genomic alignment of first-wave isolates and five VOCs showing sections of N and its 5′ region, codonized by CodAlignView in the reading frames of N (**a**) and Orf9b (**b**). The alignment includes TRS for N sgRNA present in all genomes; partial TRS for Orf9b sgRNA only in Alpha; TRS for N* sgRNA in Gamma and partial TRS in Alpha and Omicron. All mutations in Orf9b are colour-coded to indicate conservative (dark green) and radical (red) amino acid changes in Orf9b protein. We also highlighted a one-base deletion at 5′ of the N start codon in Alpha and Delta and an A to T substitution in Omicron, which change their adequate (A in −3, T in +4) Kozak initiation context to the weak (T in −3, T in +4) context, and could lead to more leaky scanning translation of Orf9b from the N sgRNA.

The SARS-CoV-2 Delta (B.1.617.2) variant of concern (VOC) contains the same noncoding deletion in the N Kozak sequence as Alpha, and the recently identified Omicron (B.1.1.529) VOC has a nucleotide substitution (28271A>T) at the same position, which would be predicted to confer a similar effect on the N Kozak context and on translation initiation (Fig. 6). Therefore, we suggest that these changes could represent key human adaptations that influence Orf9b levels, which, in turn, would dampen the immune response. Of note, the three-nucleotide change (28881–28883 GGG->AAC) that confers N* sgRNA synthesis is also present in both the Gamma (P.1/B.1.1.28.1) and the Omicron VOCs (Fig. 6). However, more work is needed to determine whether N* is involved in dsRNA sequestration or innate antagonism. Our data do not rule out coding changes in other innate antagonists being important for Alpha adaptation to humans, but highlight the need for quantitative sequencing of sgRNAs with future VOCs.

It is noteworthy that host phosphorylation regulates Orf9b activity. We hypothesize that unphosphorylated Orf9b is maximally active early after infection to permit effective innate antagonism and viral production, but that as host innate activation begins, Orf9b becomes phosphorylated and switched off, which drives subsequent innate immune activation. Such an inflammatory switch may have evolved to enhance transmission by increasing inflammation at the site of infection once virus production is high. This switch is enhanced in Alpha, as evidenced by a greater differential in Orf9b phosphorylation between early and late time points, consistent with a delayed onset of symptoms for Alpha, and enhanced inflammatory disease^43,44^. Understanding Orf9b phosphorylation mechanisms will be key to understanding this switch. We previously identified MARK1, MARK2 and MARK3 kinases as interaction partners of Orf9b^35^ and ongoing studies will reveal their role in infection and the innate response.

The importance of Alpha adaptation to avoid innate immunity is also underlined by identification of the first recombinant VOC^45^. This variant has recombined around the Orf6–Orf7 junction, combining the spike protein adaptations of enhanced entry, furin cleavage and antibody escape of the Delta variant^46–49^ with the enhanced innate immune antagonism of the Alpha variant, mediated by increased expression of N, N* and Orf9b proteins. Inter-VOC recombination is a key development in the pandemic, consistent with the known importance of recombination in the generation of coronavirus diversity^50^—in this instance linking Alpha and Delta adaptations. Our findings highlight the importance of studying changes outside the spike protein to predict the behaviour of current and future VOCs, and emphasize the importance of innate immune evasion in the ongoing process of SARS-CoV-2 adaptation to humans.

## Methods

### Cell culture

Calu-3 cells were purchased from ATCC (HTB-55) and Caco-2 cells were a gift from D. Bailey. Hela-ACE2 cells were a gift from J. E Voss^51^. HEK293T cells were a gift from J. Luban. Cells were cultured in Dulbecco’s modified Eagle’s medium (DMEM) supplemented with 10% heat-inactivated FBS (Labtech) and 100 U ml^−1^ penicillin–streptomycin, with the addition of 1% sodium pyruvate (Gibco) and 1% Glutamax. All cells were passaged at 80% confluence and they were frequently monitored for mycoplasma contamination. For infections, adherent cells were trypsinized, washed once in fresh medium and passed through a 70-µm cell strainer before seeding at 0.2 × 10^6^ cells per ml into tissue-culture plates. Calu-3 cells were grown to 60–80% confluence before infection as described previously^52^. Primary normal human bronchial/tracheal epithelial cells (ATCC PCS-300-010) were expanded at the density of 6,000 cells per cm^2^ on a layer of lethally irradiated mouse 3T3-J2 cells^53^ with keratinocyte culture medium cFAD (3:1 DMEM (Gibco) to F-12 Nut Mix (Ham) (Gibco)), 10% FBS (Sigma), 1% penicillin–streptomycin (100×, Sigma), 0.4 μg ml^−1^ hydrocortisone (Calbiochem), 5 μg ml^−1^ insulin, 10 × 10^−10^ M cholera toxin (Sigma) and 2 × 10^−9^ M triodothyronine (Sigma). Cells were stimulated with 10 ng ml^−1^ hEGF (PeproTech) at day 3 and 5 of culture. Sub-confluent cultures were trypsinized with 0.25% Trypsin-EDTA (Sigma) and seeded at 0.05 × 10^6^ cells into 0.4-μm transparent 12-well transwell inserts (Greiner) in CFAD. When cells reached confluence, basal medium was replaced with complete PneumaCult-ALI medium (StemCell) and apical medium was removed completely. Cells were cultured at the air–liquid interface for 21–24 days and basal medium was replaced every 2–3 days.

### Viruses

SARS-CoV-2 isolate VIC was provided by NISBC, and IC19, Alpha, Alpha (B) and Alpha (C) have been described previously^54^; full isolate names and GISAID references are listed below. Viruses were propagated by infecting Caco-2 cells at MOI 0.01 TCID50 per cell, in culture medium at 37 °C. Virus was collected at 72 hpi and clarified by centrifugation at 4,000 rpm for 15 min at 4 °C to remove any cellular debris. We have previously shown that infection of Caco-2 cells in these conditions does not result in activation of the innate response or cytokine carryover^52^. Virus stocks were aliquoted and stored at −80 °C. Virus stocks were quantified by extracting RNA from 100 µl of supernatant with 1 µg carrier RNA using Qiagen RNeasy clean-up RNA protocol, before measuring viral E RNA copies per ml by RT–qPCR as described below. VIC virus refers to isolate BetaCoV/Australia/VIC01/2020 and PANGO lineage B. IC19 virus refers to isolate hCoV-19/England/IC19/2020, PANGO lineage B.1.13 and GISAID accession ID EPI_ISL_475572. Alpha virus refers to isolate hCoV-19/England/204690005/2020, PANGO lineage Alpha and GISAID accession ID EPI_ISL_693401. Alpha (B) virus refers to isolate hCoV-19/England/205090256/2020, PANGO lineage Alpha and GISAID accession ID EPI_ISL_747517. Alpha (C) refers to isolate hCoV-19/England/205080610/2020, PANGO lineage Alpha and GISAID accession ID EPI_ISL_723001.

### Viral sequencing and assembly

Viral stocks were sequenced to confirm each stock was the same at consensus level to the original isolate. Sequencing was performed using a multiplex PCR-based approach using the ARTIC LoCost protocol and v3 primer set as described^55,56^. Amplicon libraries were sequenced using MinION flow cells v.9.4.1 (Oxford Nanopore Technologies). Genomes were assembled using reference-based assembly to the MN908947.3 sequence and the ARTIC bioinformatic pipeline using 20× minimum coverage cut-off for any region of the genome and 50.1% cut-off for calling single-nucleotide polymorphisms.

### Infection of human cells

For infections, MOIs were calculated using E copies per cell quantified by RT–qPCR. Cells were inoculated with diluted virus stocks for 2 h at 37 °C, subsequently washed once with PBS and fresh culture medium was added. At the indicated time points, cells were collected for analysis. For primary HAE infections, virus was added to the apical side for 2 h at 37 °C. Supernatant was then removed and cells were washed twice with PBS. All liquid was removed from the apical side and basal medium was replaced with fresh Pneumacult ALI medium for the duration of the experiment. Virus release was measured at the indicated time points by extracting viral RNA from apical PBS washes.

### Virus quantification by TCID50

Virus titres were determined by TCID50 in Hela-ACE2 cells. In brief, 96-well plates were seeded at 5 × 10^3^ cells per well in 100 µl. Eight 10-fold serial dilutions of each virus stock or supernatant were prepared and 50 µl added to four replicate wells. Cytopathic effect (CPE) was scored at 2–3 days after infection. TCID50 per ml was calculated using the Reed & Muench method, and an Excel spreadsheet created by B. D. Lindenbach was used for calculating TCID50 per ml values^57^.

### RT–qPCR of viral proteins in infected cells

RNA was extracted using RNeasy Micro Kits (Qiagen) and residual genomic DNA was removed from RNA samples by on-column DNAse I treatment (Qiagen). Both steps were performed according to the manufacturer’s instructions. cDNA was synthesized using SuperScript III with random hexamer primers (Invitrogen). RT–qPCR was performed using Fast SYBR Green Master Mix (Thermo Fisher Scientific) for host gene expression and subgenomic RNA expression or TaqMan Master mix (Thermo Fisher Scientific) for viral RNA quantification, and reactions were performed on the QuantStudio 5 Real-Time PCR systems (Thermo Fisher Scientific). Viral E RNA copies were determined by a standard curve, using primers and a Taqman probe specific for E, as described elsewhere^58^ and below. The primers used for quantification of viral subgenomic RNA are listed below; the same forward primer against the leader sequence was used for all reactions, and is as described by the Artic Network^31,55^. Using the 2^−ΔΔ*C*t^ method, sgRNA levels were normalized to GAPDH to account for differences in RNA loading and then normalized to the level of Orf1a gRNA quantified in the same way for each variant to account for differences in the level of infection. Host gene expression was determined using the 2^−ΔΔ*C*t^ method and normalized to *GAPDH* expression using the primers listed below.

The following primers and probes were used:

SARS-CoV-2 E_Sarbeco_Fwd: 5′-ACAGGTACGTTAATAGTTAATAGCGT-3′; SARS-CoV-2 E_Sarbeco_Probe1: 5′-FAM-ACACTAGCCATCCTTACTGCGCTTCG-TAMRA-3′; SARS-CoV-2 E_Sarbeco_Rev: 5′-ATATTGCAGCAGTACGCACACA-3′; 5′_Leader_Fwd: ACCAACCAACTTTCGATCTCTTGT; Orf1a_Rev: CCTCCACGGAGTCTCCAAAG; Orf6_sg_Rev:GAGGTTTATGATGTAATCAAGATTC; Orf9b_N_sgRNA_Rev: CACTGCGTTCTCCATTCTGG; S_sgRNA_Rev: GTCAGGGTAATAAACACCACGTG; Orf3a_sgRNA_Rev: GCAGTAGCGCGAACAAAATCTG; CCL2: Fwd 5′-CAGCCAGATGCAATCAATGCC-3′; Rev 5′-TGGAATCCTGAACCCACTTCT-3′; CCL3: Fwd 5′-CAGCCAGATGCAATCAATGCC-3′; Rev 5′-TGGAATCCTGAACCCACTTCT-3′; CXCL10: Fwd 5′-TGGCATTCAAGGAGTACCTC-3′; Rev 5′-TTGTAGCAATGATCTCAACACG-3′; GAPDH: Fwd5′-GGGAAACTGTGGCGTGAT-3′; Rev 5′-GGAGGAGTGGGTGTCGCTGTT-3′; IFIT1: Fwd 5′-CCTCCTTGGGTTCGTCTACA-3′; Rev 5′-GGCTGATATCTGGGTGCCTA-3′; IFIT2: Fwd 5′-CAGCTGAGAATTGCACTGCAA-3′; Rev 5′-CGTAGGCTGCTCTCCAAGGA-3′; IFNB1: Fwd 5′-AGGACAGGATGAACTTTGAC-3′; Rev 5′-TGATAGACATTGCCAGGAG-3′; IFNL1: Fwd 5′-CACATTGGCAGGTTCAAATCTCT-3′; Rev 5′-CCAGCGGACTCCTTTTTGG-3′; IFNL3: Fwd 5′-TAAGAGGGCCAAAGATGCCTT-3′; Rev 5′-CTGGTCCAAGACATCCCCC-3′; IL-6: Fwd 5′-AAATTCGGTACATCCTCGACG-3′; Rev 5′-GGAAGGTTCAGGTTGTTTTCT-3′; IL-8: Fwd 5′-ATGACTTCCAAGCTGGCCGTGGCT-3′; Rev 5′-TCTCAGCCCTCTTCAAAAACTTCTC-3′; MX1: Fwd 5′-ATCCTGGGATTTTGGGGCTT-3′; Rev 5′-CCGCTTGTCGCTGGTGTCG-3′; RSAD2: Fwd 5′-CTGTCCGCTGGAAAGTG-3′; Rev 5′-GCTTCTTCTACACCAACATCC-3′; TNF: Fwd 5′-AGCCTCTTCTCCTTCCTGATCGTG-3′; Rev 5′-GGCTGATTAGAGAGAGGTCCCTGG-3′.

### Negative-sense-specific RT–qPCR

A negative-sense-strand-specific assay for the SARS-CoV-2 E gene was designed and established. A standard reference for the E gene was generated using fragment 11 (genome positions 25,595–28,779)^59^ provided by V. Thiel. The strand-specific RNA standards were synthesized by in vitro transcription using T7 RNA polymerase, in which each RNA template is flanked with a specific non-viral sequence tag. Reverse transcription was performed using 10^10^ copies of either positive- or negative-strand RNA with or without addition of excess copies (10^7^) of the opposite strand to test the assay specificity. Negative-sense-specific qPCR reactions were performed using cDNA templates of the negative-strand templates serially diluted by 10-fold from 10^7^ to 10^2^. The qPCR reactions were conducted as follows: 95 °C for 2 min, followed by 45 cycles of 95 °C for 10 s and 60 °C for 60 s on a ViiA 7 real time PCR machine (Applied Biosystems). Results were analysed using the ViiA 7 software v.1.1 (Applied Biosystems). To evaluate the specificity of the assay, the qPCR was performed using the primers of the opposite strand side-by-side or in the presence of excess copies of the opposite strand.

### Western blot for viral proteins in infected cells

For detection of N, Orf6, spike and tubulin expression, whole-cell protein lysates were extracted with RIPA buffer, and then separated by SDS–PAGE, transferred onto nitrocellulose and blocked in PBS with 0.05% Tween 20 and 5% skimmed milk. Membranes were probed with rabbit-anti-SARS spike (Invitrogen, PA1-411-1165, 0.5 μg ml^−1^), rabbit-anti-Orf6 (Abnova, PAB31757, 4 μg ml^−1^), Cr3009 SARS-CoV-2 cross-reactive human-anti-N antibody (1 μg ml^−1^) (a gift from L. McCoy) and mouse-anti-α-tubulin (SIGMA, clone DM1A), followed by IRDye 800CW or 680RD secondary antibodies (Abcam, goat anti-rabbit, goat anti-mouse or goat anti-human). Blots were imaged using an Odyssey Infrared Imager (LI-COR Biosciences) and analysed with Image Studio Lite software.

### Flow cytometry of infected cells

For flow cytometry analysis, adherent cells were recovered by trypsinization and washed in PBS with 2 mM EDTA (PBS/EDTA). Cells were stained with fixable Zombie UV Live/Dead dye (BioLegend) for 6 min at room temperature. Excess stain was quenched with FBS-complemented DMEM. Unbound antibody was washed off thoroughly and cells were fixed in 4% PFA before intracellular staining. For intracellular detection of SARS-CoV-2 nucleoprotein, cells were permeabilized for 15 min with intracellular staining perm wash buffer (BioLegend). Cells were then incubated with 1 μg ml^−1^ CR3009 SARS-CoV-2 cross-reactive antibody (a gift from L. McCoy) in permeabilization buffer for 30 min at room temperature, washed once and incubated with secondary Alexa Fluor 488-donkey-anti-human IgG (Jackson Labs). All samples were acquired on a BD Fortessa X20 using BD FACSDiva software. Data were analysed using FlowJo v.10 (Tree Star).

### Innate immune sensing assay

HEK293T cells were seeded in 48-well plates (5 × 10^4^ cells per well) the day before transfection. For viral protein expression, cells were transfected with 100 ng of empty vector or vector encoding Orf9b, Orf9b(S50E/S53E), VIC N or Alpha N (pLVX-EF1alpha-IRES-Puro backbone), alongside 10 ng of ISG56-firefly luciferase reporter plasmid (provided by A. Bowie) and 2.5 ng of a Renilla luciferase under control of a thymidine kinase promoter (Promega), as a control for transfection. Transfections were performed with 0.75 μl fugene (Promega) and 25 μl Optimem (Gibco) per well. Cells were stimulated 24 h after plasmid transfection with the poly I:C (Invivogen), concentrations stated in the figures (final 250 μl volume per well), using Lipofectamine 2000 (Invitrogen) at a 3:1 ratio and 25 μl optimem. Cells were lysed with 100 μl passive lysis buffer (Promega) 24 h after stimulation, 30 μl of cell lysis was transferred to a white 96-well assay plate and firefly and renilla activities were measured using the Dual-Glo Luciferase Assay System (Promega), reading luminescence on a GloMax -Multi Detection System (Promega). For each condition, data were normalized by dividing the firefly luciferase activity by renilla luciferase activity and then compared to the empty-vector-transfected mock-treated control to generate a fold induction.

### Immunofluorescence staining and microscopy imaging

Cells were fixed using 4% PFA-PBS for 1h and subsequently washed with PBS. A blocking step was carried out for 1 h at room temperature with 10% goat serum/1% BSA in PBS. N protein detection was performed by primary incubation with human anti-N antibody (Cr3009, 1 μg ml^−1^) for 18 h, and washing thoroughly in PBS. Where appropriate, N protein staining was followed by incubation with mouse anti-IRF3 (sc-33641, Santa Cruz) for 1 h. dsRNA was detected by primary incubation with mouse anti-dsRNA (MABE1134, Millipore) for 18 h. Primary antibodies were detected by labelling with secondary anti-human AlexaFluor-568 and anti-mouse AlexaFluor 488 conjugates (Jackson Immuno Research) for 1 h. All cells were then labelled with either HCS CellMask DeepRed (H32721, Thermo Fisher Scientific) or Phalloidin-AlexaFluor 568 (Thermo Fisher Scientific) and Hoechst33342 (H3570, Thermo Fisher Scientific). Images were acquired using the WiScan Hermes High-Content Imaging System (IDEA Bio-Medical) at magnification 10×/0.4NA or 40×/0.75NA. Four-channel automated acquisition was carried out sequentially (DAPI/TRITC, GFP/Cy5). For the nuclear translocation assay, images were acquired at 40× magnification, 35% densityand 30% well area, resulting in 102 fields of view (FOVs) per well. For dsRNA quantification, images were acquired at 10× magnification, 100% density and 80% well area, resulting in 47 FOVs per well.

### Image analysis of immunofluorescence experiments

All image channels were pre-processed using a batch rolling ball background correction in the Fiji ImageJ software package^60^ before 514 quantification. For nuclear translocation analysis, automated image analysis was carried out using CellProfiler^61^. First, nuclei were identified as primary objects by segmentation of the Hoechst33342 channel. Cells were identified as secondary objects by nucleus-dependent segmentation of the CellMask channel. Cell cytoplasm was segmented by subtracting the nuclear objects mask from the cell masks. Nucleocapsid-positive cells were identified by identifying the nucleocapsid signal as primary objects followed by generation of a nucleocapsid mask that was then applied to filter the segmented cell population. Intensity properties were calculated for the nuclei, cytoplasm and cell object populations. Nuclear:cytoplasmic ratio was calculated as part of the pipeline by dividing the integrated intensity of the nuclei object by the integrated intensity of corresponding cytoplasm object. Plotted are 1,000 randomly sampled cells selected for each condition using the ‘Pandas’ data processing package in Python 3 with a filter of 0.1> = <5. dsRNA was quantified using the Athena software (IDEA Bio-Medical) using the ‘Intracellular Granules’ module. In short, dsRNA granules within segmented cells were thresholded on the basis of the background intensity of the mock-infected population. Infected cell populations were identified as having a minimum of two segmented dsRNA objects. For dsRNA-positive cells, intensity and area properties were calculated.

### Co-immunoprecipitation of TOM70 with Orf9b

HEK293T cells were transfected with the indicated mammalian expression plasmids using Lipofectamine 2000 (Invitrogen). Twenty-four hours after transfection, cells were collected and lysed in NP-40 lysis buffer (0.5% Nonidet P 40 Substitute (NP-40; Fluka Analytical), 50 mM Tris-HCl, pH 7.4 at 4 °C, 150 mM NaCl and 1 mM EDTA) supplemented with cOmplete mini EDTA-free protease and PhosSTOP phosphatase inhibitor cocktails (Roche). Clarified cell lysates were incubated with streptactin sepharose beads (IBA) for 2 h at 4 °C, followed by five washes with NP-40 lysis buffer. Protein complexes were eluted in the SDS loading buffer and were analysed by western blotting with the indicated antibodies. Antibodies: rabbit anti–Strep-tag II (Abcam ab232586); rabbit anti-β-actin (Cell Signaling Technology 4967); monoclonal mouse anti-Flag M2 antibody (Sigma Aldrich, F1804); and polyclonal rabbit anti-Flag antibody (Sigma Aldrich, F7425).

### Cell lysis and digestion for proteomics

Following the infection time course, cells in six-well plates were washed quickly three times in ice cold 1× PBS. Next, cells were lysed in 250 μl per well of 6M guanidine hydrochloride (Sigma) in 100 mM Tris-HCl (pH 8.0) and scraped with a cell spatula for complete collection of the sample. Samples were then boiled for 5 min at 95 °C to inactivate proteases, phosphatases and virus. Samples were frozen at −80 °C and shipped to UCSF on dry ice. On arrival, samples were thawed, an additional 250 μl per sample of 6M guanidine hydrochloride buffer was added, and samples were sonicated for 3× for 10 s at 20% amplitude. Insoluble material was pelleted by spinning samples at maximum speed for 10 min. Supernatant was transferred to a new protein lo-bind tube and protein was quantified using a Bradford assay. The entire sample (approximately 600 μg of total protein) was subsequently processed for reduction and alkylation using a 1:10 sample volume of tris-(2-carboxyethyl) (TCEP) (10 mM final) and 2-chloroacetamide (4.4 mM final) for 5 min at 45 °C with shaking. Before protein digestion, the 6M guanidine hydrochloride was diluted 1:6 with 100 mM Tris-HCl pH8 to enable the activity of trypsin and LysC proteolytic enzymes, which were subsequently added at a 1:75 (wt/wt) enzyme/substrate ratio and placed in a 37 °C water bath for 16–20 h. After digestion, 10% trifluoroacetic acid (TFA) was added to each sample to a final pH of around 2. Samples were desalted under vacuum using 50 mg Sep Pak tC18 cartridges (Waters). Each cartridge was activated with 1 ml 80% acetonitrile (ACN)/0.1% TFA, then equilibrated with 3 × 1 ml of 0.1% TFA. After sample loading, cartridges were washed with 4 × 1 ml of 0.1% TFA, and samples were eluted with 2 × 0.4 ml 50% ACN/0.25% formic acid (FA). Sixty micrograms of each sample was kept for protein abundance measurements, and the remainder was used for phosphopeptide enrichment. Samples were dried by vacuum centrifugation. The same sample was used for abundance proteomics and phosphoproteomics analysis.

### Phosphopeptide enrichment for proteomics

IMAC beads (Ni-NTA from Qiagen) were prepared by washing 3× with HPLC water, incubating for 30 min with 50 mM EDTA pH 8.0 to strip the Ni, washing 3× with HPLC water, incubating with 50 mM FeCl_3_ dissolved in 10% TFA for 30 min at room temperature with shaking, washing 3× with and resuspending in 0.1% TFA in 80% ACN. Peptides were enriched for phosphorylated peptides using a King Flisher Flex. For a detailed protocol, please contact the authors. Phosphorylated peptides were found to make up more than 90% of every sample, indicating high-quality enrichment.

### Mass spectrometry data acquisition for proteomics

Digested samples were analysed on an Orbitrap Exploris 480 mass spectrometry system (Thermo Fisher Scientific) equipped with an Easy nLC 1200 ultra-high pressure liquid chromatography system (Thermo Fisher Scientific) interfaced via a Nanospray Flex nanoelectrospray source. For all analyses, samples were injected on a C18 reverse phase column (25 cm × 75 μm packed with ReprosilPur 1.9-μm particles). Mobile phase A consisted of 0.1% FA, and mobile phase B consisted of 0.1% FA/80% ACN. Peptides were separated by an organic gradient from 5% to 30% mobile phase B over 112 min followed by an increase to 58% B over 12 min, then held at 90% B for 16 min at a flow rate of 350 nl min^−1^. Analytical columns were equilibrated with 6 μl of mobile phase A. To build a spectral library, one sample from each set of biological replicates was acquired in a data-dependent manner. Data-dependent analysis (DDA) was performed by acquiring a full scan over a *m*/*z* range of 400–1,000 in the Orbitrap at 60,000 resolving power (200 *m*/*z*) with a normalized AGC target of 300%, an RF lens setting of 40% and a maximum ion injection time of 60 ms. Dynamic exclusion was set to 60 s, with a 10-ppm exclusion width setting. Peptides with charge states 2–6 were selected for MS/MS interrogation using higher-energy collisional dissociation (HCD), with 20 MS/MS scans per cycle. For phosphopeptide-enriched samples, MS/MS scans were analysed in the Orbitrap using isolation width of 1.3 *m*/*z*, normalized HCD collision energy of 30% and normalized AGC of 200% at a resolving power of 30,000 with a 54-ms maximum ion injection time. Similar settings were used for DDA of samples used to determine protein abundance, with an MS/MS resolving power of 15,000 and a 22-ms maximum ion injection time. Data-independent analysis (DIA) was performed on all samples. An MS scan at 60,000 resolving power over a scan range of 390–1010 *m*/*z*, a normalized AGC target of 300%, an RF lens setting of 40% and a maximum injection time of 60 ms was acquired, followed by DIA scans using 8 *m*/*z* isolation windows over 400–1,000 *m*/*z* at a normalized HCD collision energy of 27%. Loop control was set to All. For phosphopeptide-enriched samples, data were collected using a resolving power of 30,000 and a maximum ion injection time of 54 ms. Protein abundance samples were collected using a resolving power of 15,000 and a maximum ion injection time of 22 ms.

### Spectral library generation and raw data processing for proteomics

Raw mass spectrometry data from each DDA dataset were used to build separate libraries for DIA searches using the Pulsar search engine integrated into Spectronaut v. 14.10.201222.47784 by searching against a database of Uniprot *Homo sapiens* sequences (downloaded 28 February 2020) and 29 SARS-CoV-2 protein sequences translated from genomic sequence downloaded from GISAID (accession EPI_ISL_406596, downloaded 5 March 2020) including mutated tryptic peptides corresponding to the variants assessed in this study. For protein abundance samples, data were searched using the default Biognosys (BGS) settings, variable modification of methionine oxidation, static modification of carbamidomethyl cysteine, and filtering to a final 1% false discovery rate (FDR) at the peptide, peptide spectrum match (PSM) and protein level. For phosphopeptide-enriched samples, BGS settings were modified to include phosphorylation of S, T and Y as a variable modification. The generated search libraries were used to search the DIA data. For protein abundance samples, default BGS settings were used, with no data normalization performed. For phosphopeptide-enriched samples, the significant post-translational modification (PTM) default settings were used, with no data normalization performed, and the DIA-specific PTM site localization score in Spectronaut was applied.

### Mass spectrometry data pre-processing

Quantitative analysis was performed in the R statistical programming language (v.3.6.1, 2019-07-05). Initial quality control analyses, including inter-run clusterings, correlations, principal component analysis (PCA), peptide and protein counts and intensities were completed with the R package artMS (v. 1.8.1). On the basis of obvious outliers in intensities, correlations and clusterings in PCA analysis, one run was discarded from the protein phosphorylation dataset (IC19 24 h replicate 2). Statistical analysis of phosphorylation and protein abundance changes between mock and infected runs, as well as between infected runs from different variants (for example, Kent versus VIC) were computed using peptide ion fragment data output from Spectronaut and processed using artMS. Specifically, quantifications of phosphorylation based on peptide ions were processed using artMS as a wrapper around MSstats, via functions artMS::doSiteConversion and artMS::artmsQuantification with default settings. All peptides containing the same set of phosphorylated sites were grouped and quantified together into phosphorylation site groups. For both phosphopeptide and protein abundance MSstats pipelines, MSstats performs normalization by median equalization, imputation of missing values and median smoothing to combine intensities for multiple peptide ions or fragments into a single intensity for their protein or phosphorylation site group, and statistical tests of differences in intensity between infected and control time points. When not explicitly indicated, we used defaults for MSstats for adjusted *P* values, even in cases of *n* = 2. By default, MSstats uses the Student’s *t*-test for *P* value calculation and the Benjamini–Hochberg method of FDR estimation to adjust *P* values. After quality control data filtering, PCA (Extended Data Fig. 3b) and Pearson’s correlation (Extended Data Fig. 3c) confirmed strong correlation between biological replicates, time points and conditions. On average, we quantified 33,000–40,000 peptides mapping to 3,600–4,000 proteins for protein abundance (Extended Data Fig. 3e), and 22,000–30,000 phosphorylated peptides mapping to 3,200–3,800 proteins (Extended Data Fig. 3f). On average we find that biological replicates had 61%–82% peptide detection overlap for protein abundance and 62%–93% phosphorylation site overlap (Extended Data Fig. 3g).

### Refining and filtering phosphorylation and abundance data

MSstats phosphorylation results had to be further simplified to effects at single sites. The results of artMS and MSstats are fold changes of specific phosphorylation site groups detected within peptides, so one phosphorylation site can have multiple measurements if it occurs in different phosphorylation site groups. This complex dataset was reduced to a single fold change per site by choosing the fold change with the lowest *P* value, favouring those detected in both conditions being compared (that is, non-infinite log_2_-transformed fold change values). This single-site dataset was used as the input for kinase activity analysis and enrichment analysis. Protein abundance data were similarly simplified when a single peptide was mapped to multiple proteins; that is, by choosing the fold change with the lowest *P* value, favouring those detected in both conditions being compared (see Supplementary Table 1 for final refined data).

### Targeted proteomics for Orf9b phosphorylation

A spectral library was constructed from the DIA data to obtain Orf9b-specific transitions. We used four proteotypic Orf9b peptides to unbiasedly assess Orf9 abundance, and for Orf9b phosphorylation we included both Ser50 (LGS(+80)PLSLNMAR) and Ser53 (LGSPLS(+80)LNMAR) and two phosphosites from heat shock proteins as internal controls for normalization and to remove any bias due to the IMAC enrichment. All samples were acquired on a Orbitrap Tribrid Lumos (Thermo Fisher Scientific) connected to a nanoLC easy 1200 (Thermo Fisher Scientific). For the whole-cell lysate samples, the peptides were separated in 50 min at 0.3 μl min^−1^ with the following gradient: 2% B (0.1% FA in MeCN) to 33% B for 40 min, followed by another linear gradient from 33% to 90% of B (1 min) and an isocratic wash at 90% was performed for kept for 10 min. Peptides were injected through self-packed columns (25 cm) packed with 1.9-μm beads (ReproSil, Waters). The column tip was kept at 2 kV and 275 °C. The mass spectrometer was operated in positive mode (OT/OT) and each MS1 scan was performed with a resolution of 120,000 at 400 *m*/*z* between 350 and 1,100 *m*/*z*. Peptide ions were accumulated for 50 ms or until the ion population reached an AGC of 5 × 10^5^. Orf9b peptides (*n* = 4) within the inclusion list were fragmented using stepped HCD with a normalized energy of 33 and a spread of ±3%. For precursor ion selection an isolation window of 1.4 Da was used and the fragments after HCD were analysed in the Orbitrap at 60,000 resolution (400 *m*/*z*). For targeted analysis of Orf9b phosphorylation we used the enriched samples with identical LC, source and MS configuration. The samples were separated in 40 min at 0.3 μl min^−1^ to concentrate the analytes in narrower peaks and increase the signal. The gradient used was from 2% B to 25% in 30 min, then B was increased to 90% in 10 min and the column was washed for 10 min. The mass spectrometer was operated in positive mode and targeted acquisition (PRM). Specifically, one MS1 scan (120,000 resolution at 400 *m*/*z*, 1 × 10^6^ AGC, 256 ms IT and mass range 500–800 *m*/*z*) was followed by four unscheduled targeted scans per cycle. An isolation width of 1.6 Dawas used per precursor and isolated peptides were fragmented using stepped HCD (33% ±3%). Each MS2 was acquired with a resolution of 60,000 and ions were accumulated for 118 ms or until reaching an AGC of 5 × 10^5^. After acquisition, each experiment was analysed separately in Skyline. Under transition settings the MS1 filter was set to count and three precursors were used (10 ppm mass error). The MS2 filtering was set to Orbitrap and the resolution was set to 60,000 (400 *m*/*z*). For the phosphorylation site experiments both b/y and a/z ions were used, whereas for the abundance experiments only y ions were included. Peaks were manually inspected for integration and boundaries refined if necessary. For Orf9b Ser50/Ser53 the presence of the proline in the peptide sequence resulted in a split chromatographic peak between the two isomers and the second peak was used for integration for all samples. For both phosphoisomers, only phophosite-specific ions were used for quantification (that is, y5-y9/b6-b10 for Ser53 and y9-y5/b2-b6 for Ser50). After export of the transition-level intensities, fragments having an S/N < 10 (for the abundance data) and an S/N < 2 (for the phosphorylation data) were removed.

### RNA quality control

Thirty total RNA samples were submitted for RNA quality control. Total RNA samples were run on the Agilent Bioanalyzer, using the Agilent RNA 6000 Nano Kit. Three samples were excluded from library preparation owing to severe degradation and/or low amounts of RNA present.

### Library preparation for RNA-seq

Twenty-seven total RNA samples were processed using the Illumina Stranded Total RNA w/Ribo-Zero Plus assay. One-hundred nanograms of each total RNA sample (quantitated on the Invitrogen Qubit 2.0 Fluorometer using the Qubit RNA HS Assay Kit) was subjected to ribosomal RNA (rRNA) depletion through an enzymatic process, which includes reduction of human mitochondrial and cytoplasmic rRNAs. After rRNA depletion and purification, RNA was primed with random hexamers for first-strand cDNA synthesis, then second-strand cDNA synthesis. During second-strand cDNA synthesis, deoxyuridine triphosphate (dUTP) was incorporated in place of deoxythymidine triphosphate (dTTP) to achieve strand specificity in a subsequent amplification step. Next, adenine (A) nucleotide was added to the 3′ ends of the blunt fragments to prevent ends from ligating to each other. The A-tail also provides a complementary overhang to the thymine (T) nucleotide on the 3′ end of the adapter. During adapter ligation and amplification, indexes and adapters were added to both ends of the fragments, resulting in 10-bp, dual-indexed libraries, ready for cluster generation and sequencing. The second strand was quenched during amplification owing to the incorporation of dUTP during second-strand cDNA synthesis, allowing for only the antisense strand to be sequenced in read 1. Thirteen cycles of amplification were performed.

### Library quality control and quantification for RNA-seq

Each library was run on the Agilent Bioanalyzer, using the Agilent High Sensitivity DNA Kit, to assess the size distribution of the libraries. They were quantitated by qPCR using a Roche KAPA Library Quantification Complete Kit (ABI Prism), and run on the Applied Biosystems QuantStudio 5 Real-Time PCR System.

### Sequencing for RNA-seq

Each library was normalized to 10 nM, then pooled equimolarly for a final concentration of 10 nM. Pooled libraries were submitted to the University of California San Francisco Center for Advanced Technology (UCSF CAT) for one lane of sequencing on the Illumina NovaSeq 6000 S4 flow cell. The run parameter was 100×10×10×100 bp.

### Viral RNA quantification from the RNA-seq dataset

Viral RNA was characterized by the junction of the leader with the downstream subgenomic sequence. Reads containing possible junctions were extracted by filtering for exact matches to the 3′ end of the leader sequence ‘CTTTCGATCTCTTGTAGATCTGTTCTC’ using the bbduk program in the BBTools package (BBTools - Bushnell B. - sourceforge.net/projects/bbmap/). This subset of leader-containing reads was left-trimmed to remove the leader, also using bbduk. The filtered and trimmed reads were matched against SARS2 genomic sequence with the bbmap program from BBtools with settings (maxindel = 100, strictmaxindel = t, local = t). The leftmost mapped position in the reference was used as the junction site. All strains were mapped against a reference SARS-Cov-2 sequence (accession NC_045512.2), except Alpha was mapped against an Alpha-specific sequence (GISAID: EPI_ISL_693401) and the resultant positions adjusted to the reference on the basis of a global alignment. Junction sites were labelled on the basis of locations of TRS sequences, or other known sites with a ± 5 base pair window as follows (genomic = 67, S = 21,553, orf3 = 25,382, E = 26,237, M = 26,470, orf6 = 27,041, orf7 = 27,385, orf8 = 27,885, N = 28,257, orf9b = 28,280, N* = 28,878). Junction reads were counted per position, a pseudocount of 0.5 was added at all positions, counts between replicates and strains were normalized to have equal ‘genomic’ reads and counts were averaged across replicate samples. Means and standard errors of counts averaged across replicates were subsequently calculated. To calculate the ratios between Alpha and VIC, counts averaged across replicates from Alpha were divided in a condition and time-point-matched manner by values from VIC or IC19. The standard error (s.e.) of the ratios was calculated as (A/B) × sqrt((s.e.A/A)² + (s.e.B/B)²).

### Host RNA analysis

All reads were mapped to the human host genome (ensembl 101) using HISAT2 aligner^62^. Host transcript abundances were estimated using human annotations (ensembl 101) using StringTie^63^. Differential gene expression was calculated on the basis of read counts extracted for each protein-coding gene using featureCount and significance was determined by the DESeq2 R package^64^. On average, we quantified 15,000–16,000 mRNA transcripts above background levels (Extended Data Fig. 3d).

### Viral protein quantification

Median normalized peptide feature (peptides with unique charge states and elution times) intensities (on a linear scale) were refined to the subset that mapped to SARS-CoV-2 protein sequences using Spectronaut (see Methods). Peptide features found in the same biological replicate (that is, owing to different elution times, for example) were averaged. Next, for each time point separately, we selected the subset of peptides that were consistently detected in all biological replicates across all conditions (no missing values), isolating the set of peptides with the best comparative potential. We then summed all peptides mapping to each viral protein for each time point separately, which resulted in our final protein intensity per viral protein per time point per biological replicate. Resulting protein intensities were averaged across biological replicates and standard errors were calculated for each condition. To calculate the ratios between Alpha and VIC, averaged intensities for Alpha were divided in a condition and time-point-matched manner by values from VIC or IC19. The standard error (s.e.) of the ratios was calculated as (A/B) × sqrt((s.e.A/A)² + (s.e.B/B)²).

### Kinase activity analysis of phosphoproteomics data

Kinase activities were estimated using known kinase–substrate relationships in the literature^65^. The resource comprises a comprehensive collection of phosphosite annotations of direct substrates of kinases obtained from six databases—PhosphoSitePlus, SIGNOR, HPRD, NCI-PID, Reactome and the BEL Large Corpus—and using three text-mining tools: REACH, Sparser and RLIMS-P. Kinase activities were inferred as a *z*-score calculated using the mean log_2_FC of phosphorylated substrates for each kinase in terms of standard error (*z* = (*M* − *u*)/s.e.), comparing fold changes in phosphosite measurements of the known substrates against the overall distribution of fold changes across the sample. A *P* value was also calculated using this approach using a two-tailed *z*-test method. This statistical approach has been previously shown to perform well at estimating kinase activities^27,66^. We collected substrate annotations for 400 kinases with available data. Kinase activities for kinases with 3 or more measured substrates were considered, leaving us with 191 kinases with activity estimates in at least 1 or more infection time points. Kinases were clustered on the basis of pathway similarity by constructing a kinase tree based on co-membership in pathway terms (from the CP (‘Canonical Pathways’) category of the Molecular Signature Database (MSigDBv7.1)).

### Pathway enrichment analysis

The pathway gene sets were obtained from the CP (that is, ‘Canonical Pathways’) category of MSigDBv7.1 (ref. ^24^). We used the same approach for this pathway enrichment analysis as we used for the kinase activity analysis. Namely, we inferred pathway regulation as *z*-score and an FDR-corrected (0.05) *P* value calculated from a *z*-test (two-tailed) comparing fold changes in phosphosite, protein abundance or RNA abundance measurements of genes designated for a particular pathway against the overall distribution of fold changes in the sample. All resulting terms were further refined to select non-redundant terms by first constructing a pathway term tree based on distances (1-Jaccard similarity coefficients of shared genes in MSigDB) between the terms. The pathway term tree was cut at a specific level (*h* = 0.8) to identify clusters of non-redundant gene sets. For results with multiple significant terms belonging to the same cluster, we selected the most significant term (that is, lowest adjusted *P* value). Next, we filtered out terms that were not significant (FDR-corrected *P* value < 0.05) for at least one contrast. Terms were ranked according to either the absolute value *z*-score across contrasts that included Alpha (see Extended Data Fig. 4a–c) or by average −log_10_(*P* values) across time-matched contrasts involving Alpha (see Fig. 2b).

### Transcription factor activity analysis

Transcription factor activities were estimated from RNA-seq data using DoRothEA^67^ which provides a comprehensive resource of transcription factor–target gene interactions and annotations indicating confidence level for each interaction on the basis of the amount of supporting evidence. We restricted our analysis to A, B and C levels that comprise the most reliable interactions. For the transcription factor activity enrichment analysis, VIPER^68^ was executed with the *t*-statistic derived from the differential gene expression analysis between variant infected and controls (wild-type) infected cells. Transcription factor activity is defined as the normalized enrichment scores (NES) derived from the VIPER algorithm. VIPER algorithm was run with default parameters except for the eset.filter parameter, which was set to FALSE and considered regulons with at least five targets.

### Selection of ISGs

ISGs were taken from a previous study^25^ and annotated as ISGs. To this list of 38 genes, we added the following based on manual curation from the literature: *IFI16*, *IFI35*, *IFIT5*, *LGALS9*, *OASL*, *CCL2*, *CCL7*, *IL6*, *IFNB1*, *CXCL10* and *ADAR*.

### Reporting summary

Further information on research design is available in the Nature Research Reporting Summary linked to this paper.

## Online content

Any methods, additional references, Nature Research reporting summaries, source data, extended data, supplementary information, acknowledgements, peer review information; details of author contributions and competing interests; and statements of data and code availability are available at 10.1038/s41586-021-04352-y.

## Supplementary information

Supplementary Figures This file contains Supplementary Figure 1: Raw western blot image for viral proteins Orf6 and N; and Supplementary Figure 2. Raw western blot image of quantification for Orf9b immunoprecipitation with TOM70.

Reporting Summary

Supplementary Table 1 Fold changes and *P* values for RNA-seq, abundance proteomics, and phosphoproteomics datasets.

Supplementary Table 2 Full pathway enrichment results of RNA-seq, abundance proteomics, and phosphoproteomics datasets (i.e. Figures 2b and Extended Data Fig. 4a-c).

Supplementary Table 3 Fold changes and p-values for interferon stimulated genes from RNA-seq and abundance proteomics datasets (i.e. Figures 2c-d).

Supplementary Table 4 Full table of calculated kinase activities for comparisons between Alpha, VIC, and IC19 (i.e. Figures 2g and Extended Data Fig. 6a).

Supplementary Table 5 Full table of calculated transcription factor activities for comparisons between Alpha, VIC, and IC19 (i.e. Figures 4a and Extended Data Fig. 6d).

Supplementary Table 6 Viral RNA and protein quantities and ratios for Alpha to VIC and IC19 (i.e. Figure 3 and Extended Data Fig. 7).

Supplementary Table 7 Read counts of subgenomic RNA mapped to SARS-CoV-2 genome (i.e. Figure 3i).

## Data Availability

Abundance proteomics and phosphoproteomics datasets have been deposited to the ProteomeXchange Consortium through the PRIDE partner repository with the dataset identifier PXD026302. Raw RNA-seq data files are available under the accession number E-MTAB-11275. Processed proteomics and RNA-seq data are available as Supplementary Information.

## Code availability

No new algorithms were developed for this project and previous algorithms used are cited in the methods.

## References

[1] Volz E (2021). Assessing transmissibility of SARS-CoV-2 lineage B.1.1.7 in England. Nature.

[2] Davies NG (2021). Estimated transmissibility and impact of SARS-CoV-2 lineage B.1.1.7 in England. Science.

[3] Galloway SE (2021). Emergence of SARS-CoV-2 B.1.1.7 lineage—United States, December 29, 2020–January 12, 2021. MMWR Morb. Mortal. Wkly Rep..

[4] Calistri P (2021). Infection sustained by lineage B.1.1.7 of SARS-CoV-2 is characterised by longer persistence and higher viral RNA loads in nasopharyngeal swabs. Int. J. Infect. Dis..

[5] Foster TL (2016). Resistance of transmitted founder HIV-1 to IFITM-mediated restriction. Cell Host Microbe.

[6] Gondim MVP (2021). Heightened resistance to host type 1 interferons characterizes HIV-1 at transmission and after antiretroviral therapy interruption. Sci. Transl. Med..

[7] Sumner RP (2017). Are evolution and the intracellular innate immune system key determinants in HIV transmission?. Front. Immunol..

[8] Zhang Q (2020). Inborn errors of type I IFN immunity in patients with life-threatening COVID-19. Science.

[9] Bastard P (2020). Autoantibodies against type I IFNs in patients with life-threatening COVID-19. Science.

[10] Pairo-Castineira E (2021). Genetic mechanisms of critical illness in COVID-19. Nature.

[11] Thorne LG (2021). SARS-CoV-2 sensing by RIG-I and MDA5 links epithelial infection to macrophage inflammation. EMBO J..

[12] Lei X (2020). Activation and evasion of type I interferon responses by SARS-CoV-2. Nat. Commun..

[13] Miorin L (2020). SARS-CoV-2 Orf6 hijacks Nup98 to block STAT nuclear import and antagonize interferon signaling. Proc. Natl Acad. Sci. USA.

[14] Hackbart M, Deng X, Baker SC (2020). Coronavirus endoribonuclease targets viral polyuridine sequences to evade activating host sensors. Proc. Natl Acad. Sci. USA.

[15] Ferrarini MG (2021). Genome-wide bioinformatic analyses predict key host and viral factors in SARS-CoV-2 pathogenesis. Commun. Biol..

[16] Sorek, M., Meshorer, E. & Schlesinger, S. Transposable elements as sensors of SARS-CoV-2 infection. Preprint at 10.1101/2021.02.25.432821 (2021).

[17] Rookhuizen, D. C., Bonte, P. E., Ye, M., Hoyler, T. & Gentili, M. Induction of transposable element expression is central to innate sensing. Preprint at 10.1101/2021.09.10.457789 (2021).

[18] Zhang L (2020). SARS-CoV-2 spike-protein D614G mutation increases virion spike density and infectivity. Nat. Commun..

[19] Hou YJ (2020). SARS-CoV-2 D614G variant exhibits efficient replication ex vivo and transmission in vivo. Science.

[20] Plante JA (2021). Spike mutation D614G alters SARS-CoV-2 fitness. Nature.

[21] Volz E (2021). Evaluating the effects of SARS-CoV-2 spike mutation D614G on transmissibility and pathogenicity. Cell.

[22] Ozono S (2021). SARS-CoV-2 D614G spike mutation increases entry efficiency with enhanced ACE2-binding affinity. Nat. Commun..

[23] Guo, K., Barrett, B. S., Mickens, K. L., Hasenkrug, K. J. & Santiago, M. L. Interferon resistance of emerging SARS-CoV-2 variants. Preprint at 10.1101/2021.03.20.436257 (2021). 10.1073/pnas.2203760119 PMC9371743 35867811

[24] Subramanian A (2005). Gene set enrichment analysis: a knowledge-based approach for interpreting genome-wide expression profiles. Proc. Natl Acad. Sci. USA.

[25] Liu H (2019). Tumor-derived IFN triggers chronic pathway agonism and sensitivity to ADAR loss. Nat. Med..

[26] Ochoa D (2016). An atlas of human kinase regulation. Mol. Syst. Biol..

[27] Hernandez-Armenta C, Ochoa D, Gonçalves E, Saez-Rodriguez J, Beltrao P (2017). Benchmarking substrate-based kinase activity inference using phosphoproteomic data. Bioinformatics.

[28] Clark K, Plater L, Peggie M, Cohen P (2009). Use of the pharmacological inhibitor BX795 to study the regulation and physiological roles of TBK1 and IκB kinase ε.. J. Biol. Chem..

[29] Heo J-M (2018). RAB7A phosphorylation by TBK1 promotes mitophagy via the PINK–PARKIN pathway. Sci. Adv..

[30] Jungreis I (2021). Conflicting and ambiguous names of overlapping ORFs in the SARS-CoV-2 genome: a homology-based resolution. Virology.

[31] Parker, M. D., Lindsey, B. B., Shah, D. R., Hsu, S. & Keeley, A. J. Altered sub-genomic RNA expression in SARS-CoV-2 B. 1.1. 7 infections. Preprint at 10.1101/2021.03.02.433156 (2021).

[32] Jungreis I, Sealfon R, Kellis M (2021). SARS-CoV-2 gene content and COVID-19 mutation impact by comparing 44 *Sarbecovirus* genomes. Nat. Commun..

[33] Oh SJ, Shin OS (2021). SARS-CoV-2 nucleocapsid protein targets RIG-I-like receptor pathways to inhibit the induction of interferon response. Cells.

[34] Schmidt, N. Novel functions of host TRIM28 in restricting influenza virus infections. Dissertation, University of Zurich (2019).

[35] Gordon DE (2020). A SARS-CoV-2 protein interaction map reveals targets for drug repurposing. Nature.

[36] Liu X-Y, Wei B, Shi H-X, Shan Y-F, Wang C (2010). Tom70 mediates activation of interferon regulatory factor 3 on mitochondria. Cell Res..

[37] Jiang H-W (2020). SARS-CoV-2 Orf9b suppresses type I interferon responses by targeting TOM70. Cell. Mol. Immunol..

[38] Gao X (2021). Crystal structure of SARS-CoV-2 Orf9b in complex with human TOM70 suggests unusual virus–host interactions. Nat. Commun..

[39] Bouhaddou M (2020). The global phosphorylation landscape of SARS-CoV-2 infection. Cell.

[40] Gordon DE (2020). Comparative host–coronavirus protein interaction networks reveal pan-viral disease mechanisms. Science.

[41] Calistri P (2021). Infection sustained by lineage B.1.1.7 of SARS-CoV-2 is characterised by longer persistence and higher viral RNA loads in nasopharyngeal swabs. Int. J. Infect. Dis..

[42] Kissler, S. M. et al. Densely sampled viral trajectories suggest longer duration of acute infection with B. 1.1. 7 variant relative to non-B. 1.1. 7 SARS-CoV-2. Preprint at 10.1101/2021.02.16.21251535 (2021).

[43] Davies NG (2021). Increased mortality in community-tested cases of SARS-CoV-2 lineage B.1.1.7. Nature.

[44] Scientific Advisory Group for Emergencies. NERVTAG: Update Note on B.1.1.7 Severity. https://assets.publishing.service.gov.uk/government/uploads/system/uploads/attachment_data/file/982640/Feb_NERVTAG_update_note_on_B.1.1.7_severity.pdf (2021).

[45] Sekizuka, T. et al. Genome recombination between Delta and Alpha variants of severe acute respiratory syndrome Coronavirus 2 (SARS-CoV-2). Preprint at 10.1101/2021.10.11.21264606 (2021). 10.7883/yoken.JJID.2021.844 35228502

[46] Saito, A. et al. Enhanced fusogenicity and pathogenicity of SARS-CoV-2 Delta P681R mutation. *Nature*10.1038/s41586-021-04266-9 (2021). 10.1038/s41586-021-04266-9 PMC8828475 34823256

[47] Mlcochova P (2021). SARS-CoV-2 B.1.617.2 Delta variant replication and immune evasion. Nature.

[48] Planas D (2021). Reduced sensitivity of SARS-CoV-2 variant Delta to antibody neutralization. Nature.

[49] Escalera, A. et al. SARS-CoV-2 variants of concern have acquired mutations associated with an increased spike cleavage. Preprint at 10.1101/2021.08.05.455290 (2021).

[50] Gribble J (2021). The coronavirus proofreading exoribonuclease mediates extensive viral recombination. PLoS Pathog..

[51] Rogers TF (2020). Isolation of potent SARS-CoV-2 neutralizing antibodies and protection from disease in a small animal model. Science.

[52] Thorne LG, Reuschl AK, Zuliani-Alvarez L (2020). SARS-CoV-2 sensing by RIG-I and MDA5 links epithelial infection to macrophage inflammation. EMBO J..

[53] Rheinwald JG, Green H (1975). Serial cultivation of strains of human epidermal keratinocytes: the formation of keratinizing colonies from single cells. Cell.

[54] Brown, J. C. et al. Increased transmission of SARS-CoV-2 lineage B.1.1.7 (VOC 2020212/01) is not accounted for by a replicative advantage in primary airway cells or antibody escape. Preprint at 10.1101/2021.02.24.432576 (2021).

[55] Meredith LW (2020). Rapid implementation of SARS-CoV-2 sequencing to investigate cases of health-care associated COVID-19: a prospective genomic surveillance study. Lancet Infect. Dis..

[56] Tyson, J. R. et al. Improvements to the ARTIC multiplex PCR method for SARS-CoV-2 genome sequencing using nanopore. Preprint at 10.1101/2020.09.04.283077 (2020).

[57] Lindenbach BD (2009). Measuring HCV infectivity produced in cell culture and in vivo. Methods Mol. Biol..

[58] Corman VM (2020). Detection of 2019 novel coronavirus (2019-nCoV) by real-time RT–PCR. Eurosurveillance.

[59] Thao TTN (2020). Rapid reconstruction of SARS-CoV-2 using a synthetic genomics platform. Nature.

[60] Schindelin J (2012). Fiji: an open-source platform for biological-image analysis. Nat. Methods.

[61] Carpenter AE (2006). CellProfiler: image analysis software for identifying and quantifying cell phenotypes. Genome Biol..

[62] Kim D, Paggi JM, Park C, Bennett C, Salzberg SL (2019). Graph-based genome alignment and genotyping with HISAT2 and HISAT-genotype. Nat. Biotechnol..

[63] Kovaka S (2019). Transcriptome assembly from long-read RNA-seq alignments with StringTie2. Genome Biol..

[64] Love MI, Huber W, Anders S (2014). Moderated estimation of fold change and dispersion for RNA-seq data with DESeq2. Genome Biol..

[65] Bachman, J. A., Gyori, B. M. & Sorger, P. K. Assembling a phosphoproteomic knowledge base using ProtMapper to normalize phosphosite information from databases and text mining. Preprint at 10.1101/822668 (2019).

[66] Casado P (2013). Kinase-substrate enrichment analysis provides insights into the heterogeneity of signaling pathway activation in leukemia cells. Sci. Signal..

[67] Garcia-Alonso L, Holland CH, Ibrahim MM, Turei D, Saez-Rodriguez J (2019). Benchmark and integration of resources for the estimation of human transcription factor activities. Genome Res..

[68] Alvarez MJ (2016). Functional characterization of somatic mutations in cancer using network-based inference of protein activity. Nat. Genet..

