# Supplementary material for: Evolution of enhanced innate immune evasion by SARS-CoV-2
Source: Nature. 2021 Dec 23;602(7897):487–95. doi: 10.1038/s41586-021-04352-y (PMC8850198; doi:10.1038/s41586-021-04352-y)
Supplement: Supplementary file 1 — This file contains Supplementary Figure 1: Raw western blot image for viral proteins Orf6 and N; and Supplementary Figure 2. Raw western blot image of quantification for Orf9b immunoprecipitation with TOM70. [file 41586_2021_4352_MOESM1_ESM.pdf]

nature portfolio

https://doi.org/10.1038/s41586-021-04352-y

Supplementary information

Evolution of enhanced innate immune evasion by SARS-CoV-2

In the format provided by the authors and unedited

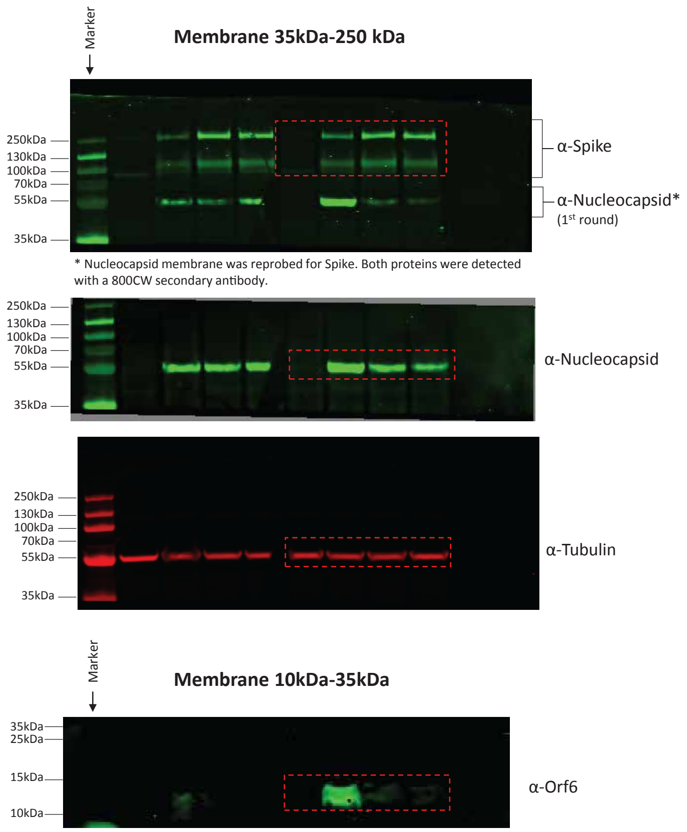

Supplementary Figure 1. Raw western blot image for viral proteins Orf6 and N. Uncropped western blot from Fig. 3f. Membrane was cut at 35kDa. Dashed lines indicate cropped area.

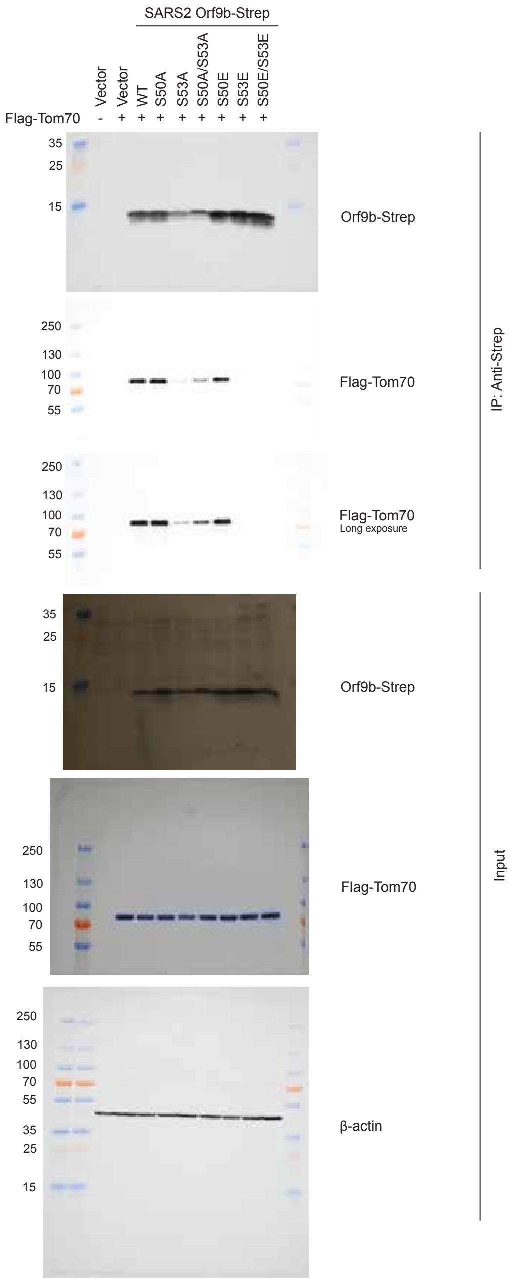

Supplementary Figure 2. Raw western blot image of quantification for Orf9b immunoprecipitation with Tom70. a. Raw western blot image corresponding to Fig. 4d or Orf9b immunoprecipitation with Tom70.
