# Supplementary material for: Evolution of enhanced innate immune evasion by SARS-CoV-2
Source: Nature. 2021 Dec 23;602(7897):487–95. doi: 10.1038/s41586-021-04352-y (PMC8850198; doi:10.1038/s41586-021-04352-y)
Supplement: Supplementary file 2 — Reporting Summary [file 41586_2021_4352_MOESM2_ESM.pdf]

nature portfolio

Corresponding author(s): Nevan Krogan
Greg Towers
Clare Jolly

Last updated by author(s): Dec 7, 2021

Reporting Summary

Nature Portfolio wishes to improve the reproducibility of the work that we publish. This form provides structure for consistency and transparency in reporting. For further information on Nature Portfolio policies, see our Editorial Policies and the Editorial Policy Checklist.

Statistics

For all statistical analyses, confirm that the following items are present in the figure legend, table legend, main text, or Methods section.

n/a	Confirmed	
☐	☒	The exact sample size (n) for each experimental group/condition, given as a discrete number and unit of measurement
☐	☒	A statement on whether measurements were taken from distinct samples or whether the same sample was measured repeatedly
☐	☒	The statistical test(s) used AND whether they are one- or two-sided<br>*Only common tests should be described solely by name; describe more complex techniques in the Methods section.*
☒	☐	A description of all covariates tested
☐	☒	A description of any assumptions or corrections, such as tests of normality and adjustment for multiple comparisons
☐	☒	A full description of the statistical parameters including central tendency (e.g. means) or other basic estimates (e.g. regression coefficient) AND variation (e.g. standard deviation) or associated estimates of uncertainty (e.g. confidence intervals)
☒	☐	For null hypothesis testing, the test statistic (e.g. F, t, r) with confidence intervals, effect sizes, degrees of freedom and P value noted<br>*Give P values as exact values whenever suitable.*
☒	☐	For Bayesian analysis, information on the choice of priors and Markov chain Monte Carlo settings
☒	☐	For hierarchical and complex designs, identification of the appropriate level for tests and full reporting of outcomes
☐	☒	Estimates of effect sizes (e.g. Cohen's d, Pearson's r), indicating how they were calculated

Our web collection on statistics for biologists contains articles on many of the points above.

Software and code

Policy information about availability of computer code

Data collection	All MS data was acquired on a Thermo Fisher Scientific Q-Exactive Plus mass spectrometer using the Thermo software Xcalibur (4.2.47) and Tune (2.11 QF1 Build 3006). RNA samples were run on an Illumina NovaSeq 6000 S4 flow cell. The run parameter was 100x10x10x100bp.
Data analysis	Raw mass spectrometry data were searched using Spectronaut whereas data normalization and quantitative comparisons were derived using the MSstats software package. RNA data was analyzed using the BBTools package (sourceforge.net/projects/bbmap/). The R statistical toolbox and GraphPad were used to perform kinase activity analysis, transcription factor activity analysis, and to generate figures.

For manuscripts utilizing custom algorithms or software that are central to the research but not yet described in published literature, software must be made available to editors and reviewers. We strongly encourage code deposition in a community repository (e.g. GitHub). See the Nature Portfolio guidelines for submitting code & software for further information.

Data

Policy information about availability of data

All manuscripts must include a data availability statement. This statement should provide the following information, where applicable:

- Accession codes, unique identifiers, or web links for publicly available datasets
- A description of any restrictions on data availability
- For clinical datasets or third party data, please ensure that the statement adheres to our policy

Abundance proteomics and phosphoproteomics datasets have been deposited to the ProteomeXchange Consortium via the PRIDE partner repository with the dataset identifier PXD026302. Reviewers may access the raw data with the username “reviewer_pxd026302@ebi.ac.uk” and password “KBANyPDu”. Raw RNAseq data files are available from the corresponding authors upon request. Processed proteomics and RNAseq data are available as supplementary information.

Field-specific reporting

Please select the one below that is the best fit for your research. If you are not sure, read the appropriate sections before making your selection.

☒ Life sciences ☐ Behavioural & social sciences ☐ Ecological, evolutionary & environmental sciences

For a reference copy of the document with all sections, see nature.com/documents/nr-reporting-summary-flat.pdf

Life sciences study design

All studies must disclose on these points even when the disclosure is negative.

Sample size	It is an accepted practice in the field of global omics technologies that biological triplicate measurements are sufficient for measuring significantly changing RNA, protein, and post-translational modifications. At least three biological replicates were independently prepared for each condition (virus and time point).
Data exclusions	Three RNA samples (across all conditions) were excluded from library preparation due to severe degradation and/or low amounts of RNA present. Two proteomics samples were excluded for poor data quality as assessed by number of detected peptides and PCA analysis.
Replication	Reproducibility between bioreplicates can be measured by the degree of variance explained by matching LC-MS feature identifications (peptide and charge) between replicates. We used standard artMS procedures. First, LC-MS features were identified and quantified by MaxQuant in each LC-MS run. Next, the strength of effect was measured as a correlation coefficient (Pearson's r) between each pair of LC-MS runs, pairing individual feature intensities between runs by their peptide and charge identifications. Correlation patterns between LC-MS runs from biological replicates are clustered along the x and y axes, showing both high correlation coefficients (near 1.0) as well as a trend for most same-bait replicates to cluster by similarity with each other, indicating consistent and bait-specific results.<br>For virus assays, all findings were replicated in a minimum of 2 distinct experiments. In addition, multiple viral isolates of Alpha were assessed to ascertain the reproducibility of results.
Randomization	The order of sample processing was randomly determined while biological replicates were run one after the other. All samples were processed and collected on the same instruments in a short time frame (roughly 3 weeks time). Therefore instrument performance did not have time to drift. QCloud was used to control instrument longitudinal performance during the project. The same procedures were applied for the RNA sequencing studies.
Blinding	Blinding is not relevant to the data because our data are acquired and processed systematically with established computational pipelines, excluding human bias. Blinding was not performed for the follow up viral infectivity experiments because blinding was not needed to remove bias.

Reporting for specific materials, systems and methods

We require information from authors about some types of materials, experimental systems and methods used in many studies. Here, indicate whether each material, system or method listed is relevant to your study. If you are not sure if a list item applies to your research, read the appropriate section before selecting a response.

Materials & experimental systems

n/a	Involved in the study	
☐	☒	Antibodies
☐	☒	Eukaryotic cell lines
☒	☐	Palaeontology and archaeology
☒	☐	Animals and other organisms
☒	☐	Human research participants
☒	☐	Clinical data
☒	☐	Dual use research of concern

Methods

n/a	Involved in the study	
☒	☐	ChIP-seq
☒	☐	Flow cytometry
☒	☐	MRI-based neuroimaging

Antibodies

Antibodies used	For detection of N, Orf6, spike and tubulin expression: rabbit-anti-SARS spike (Invitrogen, PA1-411-1165, 0.5ug/ml), rabbit-anti-Orf6 (Abnova, PAB31757, 4ug/ml), Cr3009 SARS-CoV-2 cross-reactive human-anti-N antibody (1ug/ml) (a kind gift from Dr. Laura McCoy, UCL) , mouse-anti-alpha-tubulin (SIGMA, clone DM1A) followed by IRDye 800CW or 680RD secondary antibodies (Abcam, goat anti-rabbit, goat anti-mouse or goat anti-human).<br>For Co-IP: Monoclonal mouse anti-FLAG M2 antibody (Sigma Aldrich, F1804), Polyclonal rabbit anti-FLAG antibody (Sigma Aldrich, F7425).
Validation	A negative control with no infection or overexpression of tagged protein was included in each experiment to ensure low non-specific binding of the antibodies.

Eukaryotic cell lines

Policy information about cell lines

Cell line source(s)	Calu-3 cells were purchased from ATCC (HTB-55) and Caco-2 cells were a kind gift from Dr. Dalan Bailey (Pirbright Institute, USA). Hela-ACE2 cells were a kind gift from Dr. James E Voss (TSRI, USA). HEK293T cells were a kind gift from Jeremy Luban.
Authentication	All cell lines were originally purchased from ATCC. ATCC possesses rigorous standards for cell line authentication using short-tandem repeat profiling. This confirms the identify of cells and detects misidentified, cross-contaminated, or genetically drifted cells.
Mycoplasma contamination	All cell lines are tested for mycoplasma contamination regularly, every 6 months.
Commonly misidentified lines (See ICLAC register)	No commonly misidentified cell lines were used in this study.
